# Frequency Band Analysis of Multiple Stationary Time Series

**DOI:** 10.1002/sim.70412

**Published:** 2026-02-12

**Authors:** Connor K. Brubaker, Jack P. Manning, Jennifer M. Yentes, Scott A. Bruce

**Affiliations:** ^1^ Department of Statistics Texas A&M University College Station Texas USA; ^2^ Department of Kinesiology & Sport Management Texas A&M University College Station Texas USA

**Keywords:** cluster validation, gait variability, genetic algorithm, multi‐taper estimation, spectral analysis, stride interval

## Abstract

The frequency‐domain properties of biomedical signals offer valuable insights into health and functioning of underlying physiological systems. The power spectrum, which characterizes these properties, is often summarized by partitioning frequencies into standard bands and averaging power within bands. These summary measures are regularly used for analysis in practice, but are not guaranteed to optimally retain differences in power spectra across signals from different participants. We propose a data‐adaptive method for identifying frequency band summary measures that preserve spectral variability within a population of interest. The method can also identify subpopulations with distinct power spectra and summary measures that best characterize local dynamics. Numerical selection criteria are developed to select a reasonable number of bands and subpopulations that best characterize overall dynamics. A genetic algorithm is designed to simultaneously identify subpopulations and their corresponding summary measures. The method is used to analyze stride interval series from patients with different neurological disorders, revealing distinct subpopulations and the need for subpopulation‐dependent summary measures.

## 1 Introduction

The frequency‐domain properties of biomedical signals are often correlated with underlying physiological processes and can provide valuable information about the health and functioning of an individual. The power spectrum summarizes these properties by delineating how periodic components at different frequencies contribute to the overall variance of the signal. Within a population of interest, power spectra for signals collected from different individuals often reflect differences in their physiological behavior. For example, power spectra of inter‐beat interval time series, or the time between consecutive heartbeats, are associated with activity of the parasympathetic and sympathetic branches of the autonomic nervous system [1, 2]. Power spectra of pupil diameter time series recorded during attention and working memory tasks reflect neurophysiological differences in children with and without attention deficit hyperactivity disorder (ADHD) [3, 4]. Lastly, the frequency analysis of stride interval series, or the time between consecutive steps, has shown that neurological disease is associated with greater power in higher frequencies [5, 6, 7].

To obtain a low‐dimensional summary of the power spectrum for further analysis, researchers often partition frequencies into several frequency bands and take collapsed measures of power, such as average or total power, within each band. For short‐term inter‐beat interval time series, standard frequency bands have been proposed [8, 9]. Collapsed measures of power within these bands have been used to assess the effect of stress on heart rate variability during sleep [10] and to quantify differences in heart rate variability for women among different racial groups during sleep [11]. Hemni et al. [6] used the same bands often used in the analysis of inter‐beat interval signals to analyze stride interval series and found that the severity of Parkinson's disease (PD) was strongly correlated with power in the low‐frequency range. Standard frequency bands used in the scientific literature, including those mentioned here, have been largely established via manual inspection of the data. However, these bands may not adequately summarize power spectra under all settings, which has motivated recent methodological developments providing data‐driven procedures for identifying the number and location of frequency bands for a single time series [12, 13, 14, 15].

**FIGURE 1:**
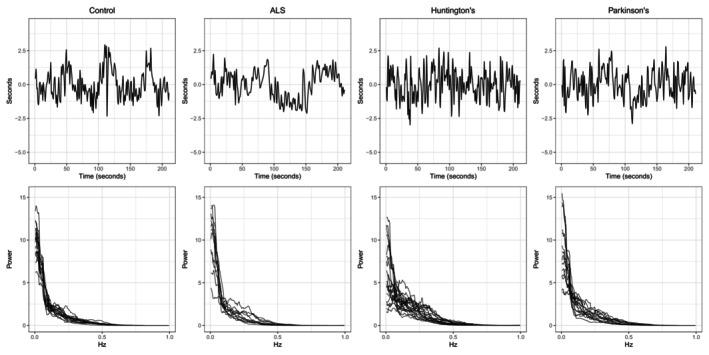
(Top) Detrended, variance‐standardized stride interval time series from four participants in the gait analysis study, one from each group that corresponds to a neurological condition: ALS, Huntington's disease, Parkinson's disease, or healthy controls. (Bottom) Estimated normalized power spectra for the detrended stride interval time series from each participant, sorted by the corresponding neurological condition in the top row.

It is common in many studies to collect time series from multiple independent subjects sampled from a population of interest [16, 17], and it is crucial that frequency band summary measures retain differences that may exist among the underlying power spectra [7, 18]. A key example and motivating application come from a study of gait and neurodegenerative disease [19]. The top row of Figure 1 shows detrended and variance‐standardized stride interval series collected from participants diagnosed with one of three neurological disorders and a group of healthy controls, and the bottom row displays all estimated normalized power spectra within each group. In addition to neurological conditions, gait is influenced by many subject‐specific characteristics such as age, neural control, and muscle function [20]. Consequently, differences exist in the spectral properties of the stride interval across both neurological conditions and among subjects with a common neurological condition. These differences can be easily lost when collapsing power spectra into frequency band summary measures. We seek to provide a data‐driven determination of the summary measures that best retain differences across members of the population.

Differences in the underlying power spectrum between subjects and the failure of traditional time series models and theory to account for it were first discussed by Diggle and Al Wasel [18]. They proposed a parametric log‐spectral mixed‐effects model to account for deviations from a population‐level power spectrum between independent time series. This model has been generalized by Iannaccone and Coles [21], who propose a nonparametric formulation of the population‐level spectrum within a mixed‐effects model, and by Krafty et al. [22], who model the effects of design covariates on the spectra while accounting for possible frequency‐domain correlation between repeated within‐subject time series segments. Lastly, Krafty [7] proposes a nonparametric model that allows for the existence of subpopulations, each with its own subpopulation‐specific marginal power spectrum, which we adopt in this article.

While these approaches excel at accounting for extra‐spectral variability between subjects, they do not immediately address the problem of determining a set of frequency band summary measures that preserve this variability. Existing methods could be adopted to determine summary measures for a single stationary process, but do not consider the setting of multiple subjects or subpopulations. For example, Granados‐Garcia et al. [23] model the power spectrum of a single stationary time series as a finite mixture of second‐order autoregressive spectra, each with its own mid‐frequency peaks and bandwidths, which could represent different frequency bands of interest. However, to the best of our knowledge, there are no existing methods that immediately offer a data‐driven determination of a set of frequency band summary measures that preserve variability across multiple spectra drawn from a population of interest.

In this article, we propose a method for the frequency band analysis of multiple time series (FBAM) in the presence of extra spectral variability, so as to preserve as much of the variability in the power spectra across the population as possible. The optimal set of frequency bands is determined by minimizing a sum of squares objective function, which can be viewed as regressing a piecewise constant function (centered at the mean power within the band) onto a collection of power spectra. We develop a validation criterion to determine the appropriate number of bands that parsimoniously summarize power spectra within the population. For heterogeneous populations comprised of subpopulations with distinctly different power spectra, such as in the gait variability study discussed above, the proposed methodology offers the ability to simultaneously identify subpopulation structures and their corresponding set of optimal frequency band summary measures. As with the number of bands, we do not assume prior knowledge of the number of subpopulations and instead develop an additional validation criterion that can be used in conjunction with the criterion for choosing the number of bands. Optimization of our objective function is a difficult task due to the size of the search space and the nonconvexity of the objective. We create a scalable genetic algorithm (GA) with a custom representation and corresponding genetic operators to efficiently traverse the parameter space and find the optimal solution.

This article makes several novel contributions. First, the proposed methodology can simultaneously identify the frequency band and subpopulation structure that best preserves spectral variability. Second, we provide validation criteria to automatically select the most appropriate number of frequency bands and subpopulations that parsimoniously summarize a population of interest without relying on knowledge of these quantities a priori. Lastly, we design and implement a computationally efficient GA that simultaneously searches partitions of time series and frequencies in a parallelized and scalable manner.

The rest of this article is organized as follows. In Section 2, we describe a model for multiple power spectra that accounts for spectral variability between and within subpopulations. In Section 3, we develop a least squares objective function and validation criteria to select the most appropriate number of frequency bands and subpopulations. In Section 4, we discuss estimation and optimization using GAs. We provide simulation studies in Section 5 to demonstrate strong finite sample performance. Finally, in Section 6, we study the application of FBAM to the analysis of stride interval series from the gait and neurological disease study.

## 2 Model

Let $\left\{X_{jkt};t\in \mathbb{Z}\right\}$ represent a collection of $k=1,\ldots ,K_{j}$ zero mean time series from subpopulation j=1,…,J. These time series are assumed to be second‐order stationary such that their variances $\mathrm{Var}[X_{jkt}]=\sigma_{jk}^{2}<\infty$ and autocovariances $E[X_{jkt_{1}}X_{jkt_{2}}]=\gamma_{jk}(t_{1}-t_{2})$ do not depend on time. We introduce a frequency‐domain stochastic transfer function model [7] to characterize the collection of time series such that

(1) $$X_{jkt}=\int_{0}^{1}A_{jk}(\omega )\exp(2\pi i\omega t)\;dZ_{jk}(\omega ).$$

This model allows for variability in the power spectra between and within subpopulations via stochastic, replicate‐specific transfer functions, $A_{jk}$, satisfying the following conditions.

Assumption 1 (Stochastic transfer functions) Each $A_{jk}$ for j=1,…,J and $k=1,\ldots ,K_{j}$ is an independent, second‐order random function identically distributed for each replicate k within subpopulation j. Any realization of $A_{jk}$ is a complex‐valued function of frequency ω that is conjugate symmetric across the origin (Hermitian) such that $A_{jk}(\omega )=\bar{A_{jk}(-\omega )}$ where $\bar{z}$ denotes the complex conjugate. Any realization $A_{jk}(\omega )$ also has period one such that $A_{jk}(\omega )=A_{jk}(\omega +1)$ for all ω. Lastly, any realization of $A_{jk}$ has uniformly continuous real and imaginary parts such that for any ε>0, there exists a δ>0 such that for all ω,λ∈ℝ, |ω−λ|<δ implies $|B_{jk}(\omega )-B_{jk}(\lambda )|<\varepsilon$ and $|C_{jk}(\omega )-C_{jk}(\lambda )|<\varepsilon$ for $A_{jk}(\omega )=B_{jk}(\omega )+iC_{jk}(\omega )$ and $i=\sqrt{-1}$.

$Z_{jk}$ are i.i.d. orthogonal increment processes that are independent of the transfer functions with $𝔼|dZ_{jk}(\omega ){|}^{2}=1$. To ensure tractable estimation, mixing conditions are assumed on the orthogonal processes $Z_{jk}$ such that $z_{jkt}=\int_{0}^{1}\exp(2\pi i\omega t)dZ_{jk}(\omega )$ is, by some measure, short‐range dependent. A time series is said to be short‐range dependent if the correlation between values at distinct time points diminishes rapidly as the lag between them increases, meaning past values have little influence on distant future values. Many different mixing conditions can be used to characterize the degree and rate of dependence between past and future values of a stochastic process. We adopt the commonly used Assumption 2.6.1 of Brillinger [24] stated below.

Assumption 2 (Mixing) For each j=1,…,J and k, $z_{jkt}=\int_{0}^{1}\exp(2\pi i\omega t)dZ_{jk}(\omega )$ is a strictly stationary time series, all of whose moments exist and satisfy $\sum_{t_{1},\ldots ,t_{q-1}=-\infty }^{\infty }|c_{jk,q}(t_{1},\ldots ,t_{q-1})|<\infty$ where $c_{jk,q}(t_{1},\ldots ,t_{q-1})=\text{cum}\{z_{jk0},z_{jkt_{1}}\ldots ,z_{jkt_{q-1}}\}$ denotes the qth order cumulant of $z_{jkt}$.

For a Gaussian process, Assumption 2 is equivalent to assuming that the autocovariance function of the process is absolutely summable over all lags. We also assume replicate‐specific spectra are smooth in the sense that their first derivatives with respect to frequency are bounded.

Assumption 3 (Smoothness) For each j=1,…,J and k, we assume $\max_{\omega \in [-1,1]}\frac{d}{d\omega }{\left|A_{jk}(\omega )\right|}^{2}<\infty$.

Each time series in the jth subpopulation is an i.i.d. stationary process with power spectrum ${\bar{g}}_{j}(\omega ):=𝔼|A_{jk}(\omega ){|}^{2}$, and $\mathrm{Var}(|A_{jk}(\omega ){|}^{2})$ measures the variability in the spectra within this subpopulation at frequency ω. Conditional on the replicate‐specific transfer function $A_{jk}(\omega )$, $X_{jkt}$ is stationary, short‐range dependent, and has replicate‐specific power spectrum $g_{jk}(\omega )=|A_{jk}(\omega ){|}^{2}$. When $\mathrm{Var}(|A_{jk}(\omega ){|}^{2})=0$ for all ω, $X_{jkt}$ is short‐range dependent and any replicate from the jth subpopulation can be used to obtain a consistent estimate of ${\bar{g}}_{j}$. However, when the variance in the replicate‐specific spectra is not trivial, which is often the case for biomedical time series [18], replicate‐specific power spectra deviate from the subpopulation‐specific expected spectrum ${\bar{g}}_{j}$ such that no single replicate can be used to consistently estimate ${\bar{g}}_{j}$.

## 3 Frequency Band Analysis

In this section, we present a method to determine the frequency band structure and summary measures that best preserve variability across a collection of power spectra, which are assumed to be realizations of the stochastic transfer function model (1). Initially, we assume the most appropriate number of subpopulations and frequency bands that best fit the data are known and fixed. Motivated by a trade‐off between model complexity and fit, validation criteria are proposed to select these quantities automatically.

### 3.1 Frequency Band Summary Measures

We begin by considering frequency bands for a particular subpopulation, and for ease of notation, temporarily omit the subpopulation index j. A set of L frequency bands is a partition $W=\{W_{1},\ldots ,W_{L}\}$ of frequency into disjoint sets $W_{1}=(0,\omega_{1}^{*})$, $W_{l}=[\omega_{l-1}^{*},\omega_{l}^{*})$ for l=2,…,L−1, and $W_{L}=[\omega_{L-1}^{*},0.5)$. Given a set of frequency bands W, summary measures of power are computed for each replicate within each band. In this article, we consider the commonly used band‐average power defined as $y_{kl}=|W_{l}{|}^{-1}\int_{W_{l}}g_{k}(\omega )\;d\omega$ where $|W_{l}|:=\omega_{l}^{*}-\omega_{l-1}^{*}$, $\omega_{0}^{*}=0$, and $\omega_{L}^{*}=0.5$. Additionally, we define the average collapsed measure of power across all replicates, $y_{\cdot l}=K^{-1}\sum_{k=1}^{K}y_{kl}$.

Given a particular number of frequency bands L, we seek to identify a set of frequency bands W that best approximates the underlying power spectra and optimally preserves the underlying spectral variability. Consequently, we define the optimal set of frequency bands as those which minimize the $L^{2}$ distance between each replicate‐specific spectrum and the resulting average collapsed measures of power in each band,

(2) $$W^{*}=\underset{W}{\text{arg min}}\;\overset{K}{\underset{k=1}{\sum }}\|g_{k}(\omega )-y(\omega ){\|}_{2}^{2}$$

where $\|g(\cdot ){\|}_{2}^{2}:=\int_{0}^{1/2}|g(u){|}^{2}\;du$ is the $L^{2}$ norm on the space of square integrable functions defined on the interval [0,1/2] and $y(\omega ):=\sum_{l=1}^{L}y_{\cdot l}1_{W_{l}}(\omega )$ where $1_{W_{l}}(\omega )=1$ if $\omega_{l-1}^{*}\leq \omega <\omega_{l}^{*}$ and 0 otherwise. y(ω) is a piecewise constant function whose breakpoints are the boundaries of the frequency bands defined by W and whose constant values are the corresponding average collapsed measures of power $y_{\cdot l}$. We refer to y(ω) as the mean collapsed power spectrum of the subpopulation.

In this case, the problem of determining the optimal set of frequency bands is equivalent to the regression of a piecewise constant function representing the average collapsed measure of power in each band onto one or more power spectra. Piecewise regression is commonly used for signal approximation [25] and change point detection [26] in univariate signals, making it a natural and appealing choice for approximating a collection of signals. If there are multiple subpopulations with known membership, this approach can be applied to each subpopulation independently. Increasing the number of bands L will typically result in a better approximation, posing a trade‐off between fit and model complexity, which we address in the next subsection.

Now, consider simultaneously modeling multiple subpopulations with unknown membership that exhibit different spectral dynamics. The objective function in (2) is generalized to determine multiple sets of subpopulation‐dependent frequency bands. We achieve this by coupling the regression above with a divisive, objective‐based clustering method. In order to facilitate comparisons across subpopulations, we make the following assumption.

Assumption 4 The number of bands L required to adequately summarize spectra within a subpopulation is the same for all subpopulations within the population under consideration.

This assumption is justified from a practical perspective in settings where we expect the power spectrum of the biomedical signal to vary across subpopulations in shape but exhibit similar complexity.

Let C be a partition of the replicate‐specific spectra into J nonempty subpopulations. Denote by $g_{jk}(\omega )$ the power spectrum of the kth replicate assigned to the jth subpopulation by C where $k=1,\ldots ,K_{j}$. Also, let $W_{j}=\{W_{j1},\ldots ,W_{jL}\}$ be the set of L frequency bands associated to the jth subpopulation. We seek to learn a partition C of subjects into J subpopulations and corresponding sets of L frequency bands $W_{1},\ldots ,W_{J}$ such that

(3) $$ℒ(C,W_{1},\ldots ,W_{J}|J,L)=\overset{J}{\underset{j=1}{\sum }}\overset{K_{j}}{\underset{k=1}{\sum }}\|g_{jk}(\omega )-y_{j}(\omega ){\|}_{2}^{2}$$

is minimized. Here $y_{j}(\omega ):=\sum_{l=1}^{L}y_{j\cdot l}1_{W_{jl}}(\omega )$ is the mean collapsed power spectrum associated to the jth subpopulation. This function is constructed using the corresponding subpopulation‐specific average collapsed measures of power $y_{j\cdot l}=K_{j}\sum_{k=1}^{K_{j}}y_{jkl}$ where $y_{jkl}=|W_{jl}{|}^{-1}\int_{W_{jl}}g_{jk}(\omega )\;d\omega$ is the replicate‐specific collapsed measure of power on the lth band for the kth replicate of the jth subpopulation. The loss function (3) is analogous to the K‐means clustering loss function. For the loss function considered here, the Euclidean norm used in the K‐means loss is substituted with the $L^{2}$ norm over square integrable functions and the cluster center is taken as the mean collapsed power spectrum $y_{j}(\omega )$, a piecewise constant approximation associated to the cluster, as opposed to the empirical average of the objects assigned to the cluster, in our case, the replicate‐specific spectra.

### 3.2 Selecting the Number of Bands and Subpopulations

We develop criteria that can be used to determine the appropriate number of frequency bands L and subpopulations J needed to adequately summarize a collection of spectra. These criteria are based on the idea of statistical similarity commonly employed in cluster validation indices used to select an appropriate number of clusters [27]. For example, the popular Davies‐Bouldin index [28] characterizes similarity between clusters as the ratio of compactness to distance. If there exist well‐separated clusters, choosing the clustering that minimizes the DB index will result in the most appropriate number of clusters for the data.

The criterion used to determine the number of frequency bands is motivated by the observation that using too many frequency bands will result in similarities among neighboring bands in terms of their average summary measures and within‐band spectral variability around these quantities. Let C be a partition of the replicate‐specific spectra into J subpopulations and $W_{j}=\{W_{j1},\ldots ,W_{jL}\}$ for j=1,…,J a set of frequency bands associated to the jth subpopulation with associated average collapsed measures $y_{j\cdot 1},\ldots ,y_{j\cdot L}$. We define the similarity between the lth and (l+1)th frequency bands for the jth subpopulation for l=1,…,L−1 as the ratio of within‐band variability to total variability across both bands,

(4) $$\begin{array}{ll} & R_{jl}^{\left(1\right)}(C,W_{1},\ldots ,W_{J}|J,L)\\ & =\frac{\begin{array}{l} {\left(\sum_{k=1}^{K_{j}}\|(g_{jk}(\omega )-y_{j\cdot l})1_{W_{jl}}(\omega ){\|}_{2}^{2}\right)}^{1/2}\\ +{\left(\sum_{k=1}^{K_{j}}\|(g_{jk}(\omega )-y_{j\cdot (l+1)})1_{W_{j,l+1}}(\omega ){\|}_{2}^{2}\right)}^{1/2} \end{array}}{{\left(\sum_{k=1}^{K_{j}}\|(g_{jk}(\omega )-\mu_{j\cdot l})1_{W_{jl}\cup W_{j,l+1}}(\omega ){\|}_{2}^{2}\right)}^{1/2}} \end{array}$$

where $\mu_{j\cdot l}$ is the average collapsed measure of power computed across the lth and (l+1)th bands, that is, $\mu_{j\cdot l}={\left(|W_{jl}|+|W_{j,l+1}|\right)}^{-1}(|W_{jl}|y_{j\cdot l}+|W_{j,l+1}|y_{j\cdot (l+1)})$. Smaller values of $R_{jl}^{\left(1\right)}$ occur when neighboring frequency bands differ substantially in average power, resulting in less variability around within‐band average power $y_{j\cdot l}$ relative to variability around average power obtained by combining bands $\mu_{j\cdot l}$. Given a set of minimizers of (3) corresponding to distinct values of L>1 but all under a fixed number of subpopulations J, we choose the solution that minimizes the average band similarity across bands and subpopulations defined as $S^{\left(1\right)}(C,W_{1},\ldots ,W_{J}|J,L)={\left(JL\right)}^{-1}\sum_{j=1}^{J}\sum_{l=1}^{L-1}R_{jl}^{\left(1\right)}(C,W_{1},\ldots ,W_{J}|J,L)$.

To identify the number of subpopulations, the similarity between the ith and jth subpopulations is defined as

(5) $$\begin{array}{ll} & R_{ij}^{\left(2\right)}(C,W_{1},\ldots ,W_{J}|J,L)\\ & \;=\frac{\begin{array}{l} {\left(K_{i}^{-1}\sum_{k=1}^{K_{i}}\|g_{ik}(\omega )-y_{i}(\omega ){\|}_{2}^{2}\right)}^{1/2}\\ +{\left(K_{j}^{-1}\sum_{k=1}^{K_{j}}\|g_{jk}(\omega )-y_{j}(\omega ){\|}_{2}^{2}\right)}^{1/2} \end{array}}{\|y_{i}(\omega )-y_{j}(\omega )){\|}_{2}} \end{array}$$

which is the ratio of the sum of within‐group spectral variability around their respective mean collapsed spectra $y_{j}(\omega )$ to the distance between their collapsed spectra. Given a set of minimizers of (3) corresponding to distinct values of J>1 but all under a fixed value of L, we choose the solution that minimizes the average maximum subpopulation similarity across bands, $S^{\left(2\right)}(C,W_{1},\ldots ,W_{J}|J,L)=J^{-1}\sum_{j=1}^{J}\max_{i\neq j}R_{ij}^{\left(2\right)}(C,W_{1},\ldots ,W_{J}|J,L)$. In the case that both J>1 and L>1 need to be selected from among a set of solutions corresponding to a set of distinct value‐pairs (J,L) that each minimize (3), we choose the solution that minimizes a weighted sum of both criteria. Further details are discussed in Section 4.3.

## 4 Estimation

### 4.1 Replicate‐Specific Power Spectra

In this section, we introduce a nonparametric estimator of the FBAM objective function (3) and of the selection criterion discussed in Section 3.2. Consider time series realizations $\left\{X_{jk1},\ldots ,X_{jkT}\right\}$ from the stochastic transfer model (1) from j=1,…,J subpopulations each containing $k=1,\ldots ,K_{j}$ series. The periodogram estimator of the kth replicate power spectrum in the jth subpopulation is $Y_{jk}(\omega_{m})=T^{-1}{\left|\sum_{t=1}^{T}X_{jkt}\exp(-2\pi i\omega_{m}t)\right|}^{2}$ where $\omega_{m}=m/T$, m=1,…,⌊T/2⌋−1 are the Fourier frequencies, ⌊n⌋ denoting the greatest integer less than or equal to n. Though asymptotically unbiased, the periodogram is not consistent [29] and exhibits bias in finite samples due to a phenomenon known as spectral leakage [30].

The multi‐taper approach [31] overcomes the limitations of the periodogram. Estimation of the spectrum from a tapered realization reduces finite sample bias, and averaging multiple tapered estimates using a collection of orthogonal tapers produces a consistent estimate. We consider use of the sine tapers $h_{rt}={\left(\frac{2}{T+1}\right)}^{1/2}\sin\left(\frac{\pi rt}{T+1}\right)$ which are a computationally efficient alternative to the standard Slepian tapers and achieve similar spectral concentration with significantly less local bias [32]. The rth direct spectral estimator is defined as the tapered periodogram under the rth data taper, ${ĝ}_{jk}^{\left(r\right)}(\omega_{m})=T^{-1}{\left|\sum_{t=1}^{T}h_{rt}X_{jkt}\exp(-2\pi i\omega_{m}t)\right|}^{2}$ for r=1,…,R. The multi‐taper estimator of the kth replicate spectrum in the jth subpopulation is the average of these direct estimators, that is, ${ĝ}_{jk}(\omega_{m})=R^{-1}\sum_{r=1}^{R}{ĝ}_{jk}^{\left(r\right)}(\omega_{m})$. This estimator is consistent if we allow R→∞ such that R/T→0 as T→∞ [33, Section 4.9], so we set $R=\lfloor T^{1/2}\rfloor$ in what follows. However, relatively fewer tapers should be used when the spectrum varies more rapidly [32], and methods for choosing R that take into account the curvature of the spectrum [34] can also be used.

### 4.2 Objective Function and Selection Criteria

The collapsed measure of power in the lth band for the kth replicate of the jth subpopulation is estimated with ${ŷ}_{jkl}=\#{\left(W_{jl}\right)}^{-1}\sum_{\omega \in W_{jl}}{ĝ}_{jk}(\omega )$, where $\#(W_{jl}):=\sum_{\omega }1_{W_{jl}}(\omega )$ is the number of Fourier frequencies contained in $W_{jl}$. The average collapsed measure of the lth band in the jth subpopulation is then ${ŷ}_{j\cdot l}=K_{j}^{-1}\sum_{k=1}^{K_{j}}{ŷ}_{jkl}$. The objective function (3) is estimated by

(6) $$\hat{ℒ}(C,W_{1},\ldots ,W_{J}|J,L)=\frac{1}{T}\overset{J}{\underset{j=1}{\sum }}\overset{K_{j}}{\underset{k=1}{\sum }}\overset{L}{\underset{l=1}{\sum }}\underset{\omega \in W_{jl}}{\sum }{\left({ĝ}_{jk}(\omega )-{ŷ}_{j\cdot l}\right)}^{2}.$$

Estimation of the selection criteria developed in Section 3.2 is carried out in a similar fashion to that of the objective function. The ratio defined in (4) is estimated as

$$\begin{array}{ll} & {\hat{R}}_{jl}^{\left(1\right)}(C,W_{1},\ldots ,W_{J}|J,L)\\ & \;=\frac{\begin{array}{l} {\left(\sum_{k=1}^{K_{j}}\sum_{\omega \in W_{jl}}{\left({ĝ}_{jk}(\omega )-{ŷ}_{j\cdot l}\right)}^{2}\right)}^{1/2}\\ +{\left(\sum_{k=1}^{K_{j}}\sum_{\omega \in W_{j,l+1}}{\left({ĝ}_{jk}(\omega )-{ŷ}_{j\cdot (l+1)}\right)}^{2}\right)}^{1/2} \end{array}}{{\left(\sum_{k=1}^{K_{j}}\sum_{\omega \in W_{jl}\cup W_{j,l+1}}{\left({ĝ}_{jk}(\omega )-{\hat{\mu }}_{jl}\right)}^{2}\right)}^{1/2}} \end{array}$$

where ${\hat{\mu }}_{jl}={\left(\#(W_{jl})+\#(W_{j,l+1})\right)}^{-1}(\#(W_{jl}){ŷ}_{j\cdot l}+\#(W_{j,l+1}){ŷ}_{j\cdot (l+1)})$. The ratio defined in (5) is estimated with

$$\begin{array}{ll} & {\hat{R}}_{ij}^{\left(2\right)}(C,W_{1},\ldots ,W_{J}|J,L)\\ & \;=\frac{\begin{array}{l} {\left(K_{i}^{-1}\sum_{k=1}^{K_{i}}\sum_{l=1}^{L}\sum_{\omega \in W_{il}}{\left({ĝ}_{ik}(\omega )-{ŷ}_{i\cdot l}\right)}^{2}\right)}^{1/2}\\ +{\left(K_{j}^{-1}\sum_{k=1}^{K_{j}}\sum_{l=1}^{L}\sum_{\omega \in W_{il}}{\left({ĝ}_{jk}(\omega )-{ŷ}_{j\cdot l}\right)}^{2}\right)}^{1/2} \end{array}}{{\left(\sum_{\omega }{\left({ŷ}_{i}(\omega )-{ŷ}_{j}(\omega )\right)}^{2}\right)}^{1/2}} \end{array}$$

where ${ŷ}_{j}(\omega )=\sum_{l=1}^{L}{ŷ}_{j\cdot l}1_{W_{jl}}(\omega )$ is the estimate of the jth subpopulation‐specific average collapsed power spectrum.

The following theorems establish the consistency of these estimators under the model and assumptions put forward in Section 2. Detailed proofs can be found in the Supporting Information.

Theorem 1 *Let* C *be a partition of observed time series* $\left\{X_{jk1},\ldots ,X_{jkT}\right\}$ *of length* T *into* j=1,…,J *subpopulations with* $k=1,\ldots ,K_{j}$ *subjects each whose underlying spectra are characterized by the stochastic transfer function model* (*1*). *Let* $W_{1},\ldots ,W_{J}$ *be sets of* L *frequency bands associated to each of the subpopulations. Then under Assumptions* *2* *and* *3*,

(7) $$\begin{array}{ll} \hat{ℒ}(C,W_{1},\ldots ,W_{J}|J,L) & =ℒ(C,W_{1},\ldots ,W_{J}|J,L)\\ & \;+{𝒪}_{P}\left(\frac{R}{T}\right)+𝒪\left(\frac{1}{T}\right). \end{array}$$

Theorem 2 *Consider the same settings and assumptions as Theorem* *1*. *Also assume that* $\sum_{k=1}^{K_{j}}\|(g_{jk}(\omega )-\mu_{j\cdot l})1_{W_{jl}\cup W_{j,l+1}}(\omega ){\|}_{2}^{2}=c_{jl}$ and $\|y_{i}(\omega )-y_{j}(\omega ){\|}_{2}^{2}=d_{ij}$ *are bounded away from 0 such that c_jl_ ≥ ε for* j=1,…,J *and* l=1,…,L−1 *and d_ij_ ≥ ε for each* i≠j for some fixed constant ε>0. *Then*

$$\begin{array}{ll} {\hat{R}}_{jl}^{\left(1\right)}(C,W_{1},\ldots ,W_{J}|J,L) & =R_{jl}^{\left(1\right)}(C,W_{1},\ldots ,W_{J}|J,L)\;\\ & \;+{𝒪}_{P}(R/T)+𝒪(T^{-1})\; \end{array}$$

*and*

$$\begin{array}{ll} {\hat{R}}_{ij}^{\left(2\right)}(C,W_{1},\ldots ,W_{J}|J,L) & =R_{ij}^{\left(2\right)}(C,W_{1},\ldots ,W_{J}|J,L)\;\\ & \;+{𝒪}_{P}(R/T)+𝒪(T^{-1}).\; \end{array}$$

The first term ${𝒪}_{P}(R/T)$ above follows from the use of multi‐taper estimates in (6) and ensures that the length of the realizations T grows faster than the number of tapers R used to estimate individual power spectra in the objective function. The second term 𝒪(1/T) follows from the approximation error resulting from the discretization of the integrals in (3). The additional assumptions for Theorem 2 require replicate‐specific spectra to exhibit nontrivial variability around the collapsed summary measure within each frequency band, and subpopulation‐specific average collapsed power spectra to be well separated, respectively. The results presented here are conditional on a fixed number of replicates. This is aligned with many practical scenarios where, for example, it is easier to collect longer time series epochs rather than to recruit new patients. The following corollary establishes subsequent consistency of the selection criterion.

Corollary 1 *Consider the same settings and assumptions as Theorem* *2*. *Then*

$$\begin{array}{ll} {Ŝ}^{\left(1\right)}(C,W_{1},\ldots ,W_{J}|J,L) & =S^{\left(1\right)}(C,W_{1},\ldots ,W_{J}|J,L)\\ & \;+{𝒪}_{P}(R/T)+𝒪(T^{-1}) \end{array}$$

*and*

$$\begin{array}{ll} {Ŝ}^{\left(2\right)}(C,W_{1},\ldots ,W_{J}|J,L) & =S^{\left(2\right)}(C,W_{1},\ldots ,W_{J}|J,L)\\ & \;+{𝒪}_{P}(R/T)+𝒪(T^{-1}). \end{array}$$

Lastly, we establish the consistency of the minimizers Ĵ and $\hat{L}$ of the selection criterion.

Theorem 3 *Consider the same settings and assumptions as Theorems* 1 *and* 2. *Let* J *and* L *be finite sets of parameter values for the number of subpopulations and number of frequency bands under consideration, respectively, such that* min(J)≥2 *and* min(L)≥2. *For convenience, write* $\theta_{\left(J,L\right)}\equiv (C,W_{1},\ldots ,W_{J})$ *where* C *is a partition of observed time series into* J *subpopulations and* $W_{j}$ *is a set of* L *frequency bands associated to the* j *th subpopulation. Define the functions* $n_{J}(\theta_{\left(J,L\right)})=J$ *and* $n_{L}(\theta_{\left(J,L\right)})=L$. *Let* $𝒢=\{{\hat{\theta }}_{\left(J,L\right)}|(J,L)\in J\times L\}$ *where* ${\hat{\theta }}_{\left(J,L\right)}=\text{arg min}ℒ(\theta |J,L)$. *Lastly, define* $Ĵ=n_{J}({\text{arg min}}_{\theta \in 𝒢}Ŝ(\theta ))$, $\hat{L}=n_{L}({\text{arg min}}_{\theta \in 𝒢}Ŝ(\theta ))$, $J^{*}=n_{J}({\text{arg min}}_{\theta \in 𝒢}S(\theta ))$, *and* $L^{*}=n_{L}({\text{arg min}}_{\theta \in 𝒢}S(\theta ))$. *Assume that* $S(\theta_{\left(J^{*},L^{*}\right)})<S(\theta )$ *for all* θ∈𝒢. *Then* $Ĵ\to_{P}J^{*}$ *and* $\hat{L}\to_{P}L^{*}$.

### 4.3 Optimization Using Genetic Algorithms

The objective function $\hat{ℒ}(C,W_{1},\ldots ,W_{J}|J,L)$ is generally nonsmooth and multimodal, so its optimization is not an easy task. We propose using a GA to overcome this challenge. EAs are a broad class of optimization algorithms that include the GA [35, 36], and particle swarm optimization [37]. EAs are inspired by the principles of the Darwinian theory of evolution and differ from traditional optimization procedures commonly used by statisticians (e.g., gradient descent) in two primary ways. First, a collection of possible solutions, as opposed to a single solution, is maintained at each iteration of the algorithm. Second, the selection of and subsequent modifications to these solutions for the next iteration are done stochastically. For a broad overview of EAs, see Eiben and Smith [38].

Our implementation of the GA begins with the initialization of a population of candidate solutions. These vectors, known as *chromosomes*, are allowed to “evolve” in the following way. Each chromosome produces several offspring via a *mutation* operation. These offspring are then ranked according to their fitness so that those with lower values of the objective function $\hat{ℒ}$ are given a higher rank. Finally, the best of these individuals are kept to form the next generation. This process is repeated until some convergence criteria are satisfied.

In one application of the mutation operation to a chromosome, one offspring chromosome is produced. Each entry of the original chromosome is modified with probability $p_{m}$. This operation helps prevent the algorithm from becoming stuck in a local minimum. To guarantee monotonicity of the algorithm, an *elitist* step is also performed at the end of each generation where the best chromosome of the previous generation, known as the *elite*, replaces the worst chromosome of the current generation.

Due to the possible multimodality of the objective function $\hat{ℒ}$, a single population of chromosomes can become stuck in a local minimum, which leads to premature convergence. To overcome this, we implement the island model where I populations are subject to evolution in parallel. Periodically, a number of individuals are migrated among the populations according to some migration policy. In our GA, after every M generations, a random set of N chromosomes from the (i−1)th population replaces the same number of randomly selected chromosomes in the ith population for i=2,…,I. For i=1, N randomly selected chromosomes from the Ith population replace those in the first population that were sent to the second population. In our simulations, we use I=6 islands, M=50, N=5, and a population size of 50.

When the number of subpopulations J and frequency bands L are assumed known and fixed a priori, a single GA as described above is used to optimize $\hat{ℒ}$. When they are unknown, we use this GA to minimize the objective function over a grid of possible values for J∈J and L∈L, which can be done in parallel. The solution that minimizes the selection criterion discussed in Section 3.2 is chosen as the final solution. Since there is no guarantee that the two criteria ${Ŝ}^{\left(1\right)}$ and ${Ŝ}^{\left(2\right)}$ will be of similar magnitude, we scale them in the following manner. For convenience, write $\theta_{\left(J,L\right)}=(C,W_{1},\ldots ,W_{J})$ and define $𝒢=\{{\hat{\theta }}_{\left(J,L\right)}|(J,L)\in J\times L\}$ where ${\hat{\theta }}_{\left(J,L\right)}=\text{arg min}\hat{ℒ}(\theta |J,L)$. Set $a_{1}=\sup_{\theta \in 𝒢}{Ŝ}^{\left(1\right)}(\theta |J,L)$ and $a_{2}=\sup_{\theta \in 𝒢}{Ŝ}^{\left(2\right)}(\theta |J,L)$. Then the solution θ∈𝒢 that minimizes $a_{1}^{-1}{Ŝ}^{\left(1\right)}(\theta |J,L)+a_{2}^{-1}{Ŝ}^{\left(2\right)}(\theta |J,L)$ is taken as the final solution. Scaling in this manner ensures that no one criterion influences the choice of solution more than the other since the choice of J and L is equally important. Full details of the GA and a discussion of the effect of unequal weighting of these criteria are discussed in the Supporting Information.

## 5 Simulation Studies

**FIGURE 2:**
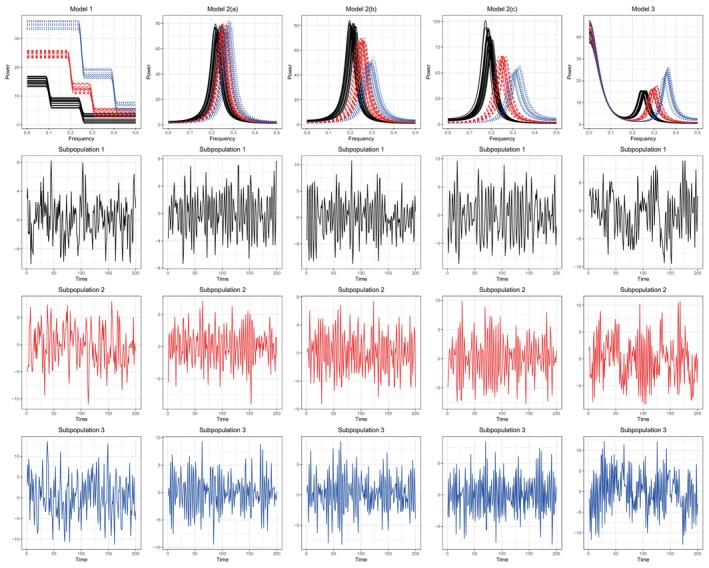
(Top) Underlying spectra from a sample realization of each model. (Bottom) A representative time series realization from each subpopulation from the corresponding model in the top row.

To evaluate the performance of FBAM, we consider three general simulation settings. Figure 2 shows realizations from each model with 20 replicates per subpopulation. The first model is defined by three subpopulations of time series whose underlying power spectra are piecewise smooth [39]. These spectra resemble piecewise constant functions except that the transitions from one level to the next are continuous. The motivation for this model is to provide a data setting that has a very clear frequency band structure and associated average summary measures within each band. The kth replicate in the jth subpopulation has underlying power spectrum

$$g_{jk}(\omega )=\overset{3}{\underset{l=1}{\sum }}y_{jkl}1_{W_{jl}}(\omega )+\overset{2}{\underset{l=1}{\sum }}\xi_{jkl}(\omega )1_{W_{jl}^{\prime }}(\omega )$$

where $\xi_{jkl}(\cdot )$ is a cubic spline on the interval $W_{jl}^{\prime }=[\omega_{jl}^{*}-0.025,\omega_{jl}^{*}+0.025)$ that connects the levels of spectrum at $y_{jkl}$ and $y_{jk(l+1)}$. The level of the spectrum on the interval $W_{jl}$ is given by $y_{jkl}∼U[y_{j\cdot l}-2,y_{j\cdot l}+2]$ independently of all other replicates where $W_{j1}=[0,\omega_{jl}^{*}-0.025)$, $W_{j2}=[\omega_{j1}^{*}+0.025,\omega_{j2}^{*}-0.025)$, and $W_{j3}=[\omega_{j2}^{*}+0.025)$ for each j=1,2,3. We set $\omega_{11}^{*}=0.1$, $\omega_{12}^{*}=0.25$, $\omega_{21}^{*}=0.2$, $\omega_{22}^{*}=0.3$, $\omega_{31}^{*}=0.25$, and $\omega_{32}^{*}=0.4$. Lastly, we set $y_{1\cdot 1}=15$, $y_{1\cdot 2}=7.5$, $y_{1\cdot 3}=2$, $y_{2\cdot 1}=25$, $y_{2\cdot 2}=12.5$, $y_{2\cdot 3}=4$, $y_{3\cdot 1}=35$, $y_{3\cdot 2}=17.5$, and $y_{3\cdot 3}=6$. Time series realizations were simulated using Theorem 3.1 of Guo and Dai [40].

The second model is defined by three subpopulations of second‐order autoregressive processes $X_{jkt}=\phi_{jk1}X_{jk(t-1)}+\phi_{jk2}X_{jk(t-2)}+\varepsilon_{jkt}$ where $\varepsilon_{jkt}∼𝒩(0,2.25^{2})$. The autoregressive parameters $\phi_{jk1}$ and $\phi_{jk2}$ are selected in such a way that the power spectra have mid‐frequency peaks. We borrow the parameterization discussed in Granados‐Garcia et al. [23] where the autoregressive parameters are written in terms of the peak location $\psi_{jk}$ and corresponding bandwidth $L_{jk}$ in the underlying spectrum, that is, $\phi_{jk1}=2\cos(2\pi \psi_{jk})\exp(-L_{jk})$ and $\phi_{jk2}=-\exp(-2L_{jk})$ where $\psi_{jk}∼U[\psi_{j}-0.02,\psi_{j}+0.02]$. We consider three special cases of this model, which correspond to decreasing levels of overlap between each subpopulation of spectra, which we name Models 2(a), 2(b), and 2(c), respectively. For Model 2(a), we set $\psi_{1}=0.23$, $\psi_{2}=0.25$, and $\psi_{3}=0.27$ and set $L_{jk}∼U[0.15-0.005,0.15+0.005]$ for each j=1,2,3. For Model 2(b), we set $\psi_{1}=0.21$, $\psi_{2}=0.25$, and $\psi_{3}=0.29$ and set $L_{2k}∼U[0.15-0.005,0.15+0.005]$ and $L_{jk}∼U[0.155-0.005,0.155+0.005]$ for j=1,3. Lastly, for Model 2(c), we set $\psi_{1}=0.19$, $\psi_{2}=0.25$, and $\psi_{3}=0.31$ and set $L_{2k}∼U[0.15-0.005,0.15+0.005]$ and $L_{jk}∼U[0.165-0.005,0.165+0.005]$ for j=1,3.

Lastly, Model 3 is defined by three subpopulations of time series that are mixtures of two independent second‐order autoregressive process which are both parameterized in the same manner as the previous model. This model provides a setting where spectra across subpopulations differ in only part of the frequency range. Define $X_{1,jkt}=\phi_{11}X_{1,jk(t-1)}+\phi_{12}X_{1,jk(t-2)}+\varepsilon_{1,jkt}$ and $X_{2,jkt}=\phi_{21}X_{2,jk(t-1)}+\phi_{22}X_{2,jk(t-2)}+\varepsilon_{2,jkt}$ where $\varepsilon_{1,jkt}∼𝒩(0,2.5^{2})$ and $\varepsilon_{2,jkt}∼𝒩(0,2^{2})$ for j=1,2,3 and $k=1,\ldots ,K_{j}$. The kth time series in the jth subpopulation is $X_{jkt}=X_{1,jkt}+X_{2,jkt}$ where the parameters of $X_{1,jkt}$ have been chosen to create a broad low‐frequency (LF) peak and those of $X_{2,jkt}$ have been chosen to create three distinct mid‐frequency peaks corresponding to the three subpopulations. Specifically, we write $\phi_{n,1}=2\cos(2\pi \psi_{n,jk})\exp(-L_{n,jk})$ and $\phi_{n,2}=-\exp(-2L_{n,jk})$ for n=1,2 and set $\psi_{1,jk}=0$ and $L_{1,jk}∼U[0.5-0.02,0.5+0.02]$ for all j and k and set $\psi_{2,1k}∼U[0.2-0.015,0.2+0.015]$, $\psi_{2,2k}∼U[0.26-0.015,0.26+0.015]$, $\psi_{2,3k}∼U[0.32-0.015,0.32+0.015]$ and $L_{2,1k}=0.05$, $L_{2,2k}=0.065$, and $L_{2,3k}=0.095$.

We fit FBAM on 100 independent realizations of each of the five models described above for each combination of time series length T∈{250,500,1000} and replicates per subpopulation $K_{j}\in \{10,20,30\}$. For each repetition, the solution that minimized the joint selection criterion over the parameter grid (J,L)∈{2,…,6}×{2,…,6} was chosen as the final solution. To evaluate the ability of FBAM to discover subpopulations of interest, we report the Adjusted Rand Index (ARI) [41] between the known subpopulation memberships defined by each model and those estimated by FBAM (Table 1). To evaluate the performance of the selection criteria, we also report in Table 1 the mean and standard deviation of the values of J and L across the final solutions.

**TABLE 1:** Mean (standard deviation) of the ARI, the number of subpopulations J, and frequency bands L of the final solutions.

Model	$K_{j}$	T	ARI	J	L
Model 1	10	250	0.76 (0.25)	2.58 (0.55)	2.30 (0.46)
		500	0.98 (0.09)	2.96 (0.20)	2.78 (0.42)
		1000	1.00 (0.04)	2.99 (0.10)	2.93 (0.26)
	20	250	0.77 (0.24)	2.56 (0.50)	2.20 (0.40)
		500	0.98 (0.08)	2.98 (0.20)	2.85 (0.36)
		1000	1.00 (0.00)	3.00 (0.00)	2.94 (0.24)
	30	250	0.82 (0.22)	2.68 (0.47)	2.17 (0.38)
		500	1.00 (0.01)	3.00 (0.00)	2.86 (0.35)
		1000	1.00 (0.00)	3.00 (0.00)	2.98 (0.14)
Model 2(a)	10	250	0.22 (0.09)	4.63 (1.04)	3.00 (0.00)
		500	0.27 (0.10)	4.10 (1.09)	3.00 (0.00)
		1000	0.29 (0.11)	3.92 (0.97)	3.00 (0.00)
	20	250	0.22 (0.08)	4.46 (1.05)	3.00 (0.00)
		500	0.27 (0.07)	3.97 (0.97)	3.00 (0.00)
		1000	0.30 (0.07)	3.90 (0.90)	3.00 (0.00)
	30	250	0.23 (0.07)	4.40 (1.23)	3.00 (0.00)
		500	0.28 (0.07)	3.94 (1.01)	3.00 (0.00)
		1000	0.29 (0.05)	3.72 (0.70)	3.00 (0.00)
Model 2(b)	10	250	0.55 (0.14)	4.67 (1.01)	3.00 (0.00)
		500	0.59 (0.15)	4.57 (0.97)	3.00 (0.00)
		1000	0.63 (0.14)	4.44 (0.84)	3.00 (0.00)
	20	250	0.55 (0.13)	4.44 (0.91)	3.00 (0.00)
		500	0.58 (0.12)	4.37 (0.82)	3.00 (0.00)
		1000	0.59 (0.12)	4.70 (0.89)	3.00 (0.00)
	30	250	0.57 (0.14)	4.36 (1.02)	3.00 (0.00)
		500	0.59 (0.11)	4.57 (0.84)	3.00 (0.00)
		1000	0.59 (0.10)	4.73 (0.79)	3.00 (0.00)
Model 2(c)	10	250	0.80 (0.17)	4.23 (1.14)	3.00 (0.00)
		500	0.87 (0.14)	3.87 (0.95)	3.00 (0.00)
		1000	0.87 (0.15)	3.92 (1.03)	3.00 (0.00)
	20	250	0.89 (0.13)	3.60 (0.88)	3.00 (0.00)
		500	0.89 (0.13)	3.72 (0.90)	3.00 (0.00)
		1000	0.86 (0.14)	4.02 (1.05)	3.00 (0.00)
	30	250	0.90 (0.12)	3.50 (0.81)	3.00 (0.00)
		500	0.92 (0.12)	3.54 (0.82)	3.00 (0.00)
		1000	0.89 (0.13)	3.80 (0.97)	3.00 (0.00)
Model 3	10	250	0.88 (0.14)	3.23 (0.63)	4.00 (0.00)
		500	0.98 (0.06)	3.07 (0.29)	4.00 (0.00)
		1000	0.98 (0.05)	3.11 (0.35)	4.00 (0.00)
	20	250	0.94 (0.09)	3.08 (0.37)	4.00 (0.00)
		500	0.98 (0.04)	3.07 (0.26)	4.00 (0.00)
		1000	0.99 (0.03)	3.04 (0.20)	4.00 (0.00)
	30	250	0.94 (0.07)	3.08 (0.34)	4.00 (0.00)
		500	0.99 (0.01)	3.00 (0.00)	4.00 (0.00)
		1000	1.00 (0.02)	3.02 (0.14)	4.00 (0.00)

The mean ARI (and its standard deviation) across the chosen solutions generally increases (decreases) with T and $K_{j}$ for Models 1 and 3, which both have well‐separated subpopulations. This indicates strong performance with respect to subpopulation discovery. However, in some cases mean ARI decreases slightly with increasing $K_{j}$ under fixed T when T is small. This is not surprising since T must be large to achieve consistent estimation (see Theorem 1) and since some replicate‐specific spectra may resemble spectra of other subpopulations by chance [7]. For Model 2, as the separability in the spectra increases, the maximum mean ARI improves. The chosen value of J stabilizes toward the number of subpopulations defined by each model with increasing T and $K_{j}$, although FBAM tends to overestimate the number of subpopulations for Model 2 due to the lack of separability and relative within‐subpopulation variability in the underlying spectra. This suggests that as long as the subpopulations in the data are well separated, FBAM can distinguish between them. Across all models, the estimated number of frequency bands L quickly stabilizes toward a reasonable value that parsimoniously represents the dynamics of the spectra within each subpopulation.

**FIGURE 3:**
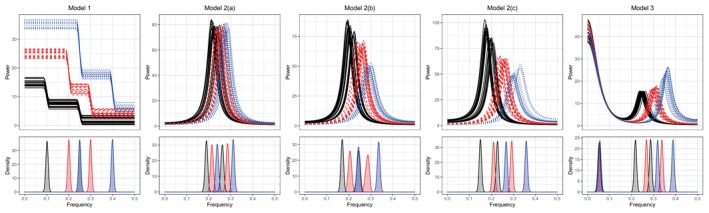
Distribution of estimated frequency band boundaries colored according to subpopulation assignment within the corresponding model in the top row. Here, T=500, $K_{j}=20$, J=3 and L=3 for Model 1 and 2(a) through 2(c) and L=4 for Model 3.

To illustrate the estimated frequency band structures, Figure 3 displays the estimated frequency band boundaries for each subpopulation across the 100 repetitions for each model when T=500, $K_{j}=20$, J=3 and L=3 for Model 1 and 2(a) through 2(c) and L=4 for Model 3. The estimated endpoints are sorted according to subpopulation label after being best matched to those defined by the model. The density plots of these estimated endpoints in Figure 3 clearly show for each model that the estimated frequency bands suitably and parsimoniously summarize the dynamics of the underlying spectra in each subpopulation. Additionally, these estimates are consistent across independent realizations of each model within each subpopulation.

While there are no existing methods that immediately offer frequency band and subpopulation estimation, we have constructed an alternative ad‐hoc approach for comparison. This begins with a functional clustering algorithm [42] to partition replicate‐specific spectra into a user‐specified number of subpopulations, followed by an application of the Bayesian mixture autoregressive decomposition (BMARD) [23]. BMARD approximates replicate‐specific spectra as a mixture of AR(2) processes, and additional post‐processing steps are taken to obtain a frequency partition from the AR(2) mixture representation. Full details of this ad‐hoc alternative method can be found in the Supporting Information.

Table 2 summarizes the results of this comparison when $K_{j}=20$ and T=500. We present the ARIs, a selected number of bands, the estimated least squares objective functions (6), and runtime ratios. Mean ARI achieved in each model by FBAM is comparable to that of the functional clustering method. Across all models, the alternative method estimates a greater number of frequency bands compared to FBAM. From an approximation and summarization viewpoint, our method is favorable since it achieves a more faithful representation of the data (i.e., smaller $\hat{ℒ}$) with fewer bands. Finally, computation time for the alternative method was 4 to 8 times longer on average, indicating significant computational savings with our simultaneous estimation procedure.

**TABLE 2:** Mean (standard deviation) of the adjusted Rand Index, selected number of bands, optimized objective function values for both FBAM and alternative method, and the ratio of execution times for the alternative method to that of FBAM.

	Adjusted Rand Index		Selected number of bands L		Optimized objective ${\hat{ℒ}}^{*}$		Runtime
	FBAM	Alternative	FBAM	Alternative	FBAM	Alternative	Ratio
**M1**	0.98 (0.08)	1.00 (0.01)	2.85 (0.36)	6.95 (0.19)	10.39 (1.79)	13.77 (0.83)	8.88 (1.12)
**M2(a)**	0.27 (0.07)	0.31 (0.07)	3.00 (0.00)	7.21 (0.27)	34.01 (4.72)	45.44 (5.22)	5.58 (2.70)
**M2(b)**	0.58 (0.12)	0.77 (0.14)	3.00 (0.00)	7.21 (0.22)	32.68 (1.59)	39.50 (5.11)	5.47 (1.02)
**M2(c)**	0.89 (0.13)	0.99 (0.03)	3.00 (0.00)	7.22 (0.23)	36.70 (2.61)	42.62 (5.28)	6.16 (0.53)
**M3**	0.98 (0.04)	0.99 (0.02)	4.00 (0.00)	5.98 (0.37)	40.33 (2.73)	65.45 (8.32)	4.42 (0.68)

## 6 Application to Gait Variability

The analysis of gait can provide insight into how different neurological conditions affect systems that regulate walking. Gait is often measured by the duration of the gait cycle, also known as the stride interval, which measures the time between contact with the ground of one foot and the repeated contact with the ground of that same foot in normal walking. Gait variability refers to the natural variation in the stride interval from one step to the next. Patients with neurodegenerative disease often exhibit different patterns of variability in gait as compared to healthy individuals [5, 19]. Higher variability in gait is reflected in the power spectrum of patients with PD, for example, through greater power in higher frequencies compared to that of a healthy individual [6].

In this section, we apply FBAM to the stride interval data [19]. The study included participants who were diagnosed with one of the following neurological conditions: amyotrophic lateral sclerosis (ALS), Huntington's disease (HD), or PD. A cohort of healthy controls was also included in the study. These data are publicly available from PhysioNet [19, 43]. Stride intervals were determined for each participant using a pressure sensor fitted to the soles of each participant who were asked to walk at a normal pace along a 77‐meter‐long hallway for 5 min. Our analysis considers 3.5 min of stride intervals defined by the left foot after a 20‐second warm‐up period. Outliers were identified as intervals outside the 1st and 96th percentiles of each signal and were imputed using a weighted moving average model [44]. Linear interpolants of the stride intervals were sampled at 2 Hz, linear trends were removed, and each signal was standardized after application of a high‐pass Butterworth filter to remove ultra‐low frequencies. The resulting data are of length T=420, which represents 210 s of walking time. This analysis considers n=61 participants: 16 healthy controls, 11 ALS participants, 20 HD participants, and 14 PD participants. Multi‐taper estimates of the normalized spectral densities of each time series were estimated using R=20 sine tapers.

**FIGURE 4:**
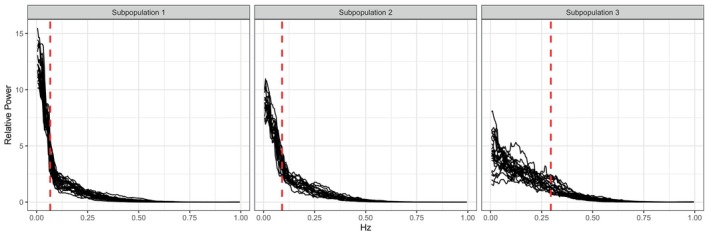
Multi‐taper estimated normalized power spectra (see Section 1) sorted by subpopulation along with the associated frequency bands determined under the second mode of summarization described in the text.

The goal of this analysis is to first identify a set of frequency bands that best characterizes the entire population and second, to simultaneously identify subpopulations with distinct patterns of gait variability, along with the subpopulation‐specific frequency bands that capture those differences. For the former, FBAM selected two frequency bands, separated at the boundary $\omega_{1}^{*}=0.081$ Hz. For the latter, FBAM identified three subpopulations, with two bands associated with each with boundaries $\omega_{11}^{*}=0.07$ Hz, $\omega_{21}^{*}=0.09$ Hz, and $\omega_{31}^{*}=0.3$ Hz. The subpopulation‐specific mean collapsed power spectra capture approximately 81% of the total variability in the spectra (see Figure 4).

**FIGURE 5:**
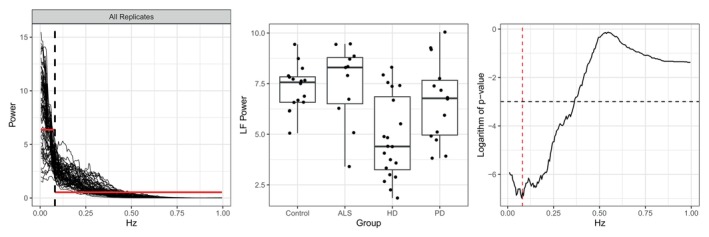
(Left) All 61 multi‐taper estimates along with the frequency bands and average summary measures suggested by FBAM to characterize the entire population. (Middle) Low‐frequency (LF) replicate‐specific collapsed measures of power by neurological condition computed using the frequency bands shown in the left plot. (Right) Logarithm of Kruskal–Wallis p‐value as a function of frequency band boundary. The dashed horizontal line corresponds to p=0.05, and the solid vertical line marks the FBAM boundary seen in the left plot.

Researchers often use frequency band collapsed measures of power to evaluate group differences, making it crucial that FBAM preserves any existing differences between groups. Visual distinctions between groups are evident when examining the stride interval power spectra in Figure 1. Here, we show that FBAM successfully retains these differences in the resulting collapsed power measures. For each potential boundary between two bands, we compute LF summary measures for all replicates using this boundary. Group differences are quantified using the Kruskal–Wallis rank sum test. Figure 5 demonstrates that the frequency band boundary automatically determined by FBAM nearly optimally preserves these differences, as reflected in the Kruskal–Wallis p‐value.

We now analyze the three subpopulations identified by FBAM, whose power spectra differ with respect to the relative magnitude and spread of low‐frequency power. Disease group and covariate summaries within each are given in Table 3. Subpopulations 1 and 2 include 15 of the 16 control patients, while 14 of the 20 HD patients are placed into subpopulation 3. ALS patients with spectra similar to controls are in subpopulations 1 and 2, whereas the two ALS patients in subpopulation 3 show the most deviation from the ALS group. PD patients are almost evenly split among the subpopulations due to high spectral variability. A large factor contributing to differences across subpopulations is related to apparent differences in the handling of turns.

**TABLE 3:** Disease group membership and median (median absolute deviation) of each of the considered covariates across the two subpopulations estimated by FBAM.

	Control	ALS	HD	PD	Age	Height	Mass	Gait speed	Severity
j=1	7	6	4	5	46.91 (17.07)	1.82 (0.12)	72.05 (13.32)	1.19 (0.20)	0.17 (0.24)
j=2	8	3	2	4	48.24 (18.00)	1.82 (0.10)	69.90 (14.40)	1.18 (0.31)	0.20 (0.26)
j=3	1	2	14	5	55.86 (15.48)	1.83 (0.12)	72.22 (18.32)	1.12 (0.29)	0.46 (0.27)

The effects of turns at the end of the hallway remain present in the detrended time series, particularly in healthy control patients, which are visible at approximately 50, 110, and 170 s in the top left of Figure 1. It has been observed that younger, healthier adults often prefer a pivot or spin turn strategy, leading to slightly longer stride intervals during turns compared to regular walking. On the other hand, older adults with turning difficulties associated with impaired balance control during gait typically prefer slower turns with more steps, more closely resembling regular walking patterns [45, 46]. Pivot turning leads to higher and more tightly concentrated relative power in the LF range compared to step turning, which distinguishes the three subpopulations detected by FBAM.

Consistent with these turning strategy differences, subpopulations also differ in disease severity (standardized so that 0 represents a healthy control and 1 the worst‐case score within each condition). While Kruskal–Wallis tests found no differences in age (p=0.19), height (p=0.85), mass (p=0.83), or average gait speed (p=0.55), disease severity was found to be unequal across the three subpopulations ($p=5.63\times 10^{-4}$). Subpopulation 3 consists of patients with more advanced disease compared to subpopulations 1 and 2. The increased severity in this group aligns with a reliance on step turning strategies, as patients with greater impairment prioritize stability during turns to mitigate fall risk. This leads to relatively less low‐frequency power and a greater spread among a wider range of low frequencies in this subpopulation. This suggests that different frequency bands may be needed when analyzing spectra for populations with different levels of disease progression.

To validate the informative power of the frequency band summary measures, we assess their ability to identify the presence of HD from among all other disease groups considered in this study. To perform this binary classification task, we employ a logistic regression model and use 6‐fold cross‐validation to estimate the out‐of‐sample classification accuracy. Our baseline model includes age, height, and gait speed and achieves 57.42% (s.e. 0.08%) accuracy. Our expanded model considers the LF summary measures derived from the frequency bands estimated for the entire population and achieves an accuracy of 78.80% (s.e. 0.09%). Further, we estimated the accuracy of the set of models where each model uses a distinct set of LF summary measures derived from each of the possible boundaries for the LF band. We found that the model that uses the LF measures derived from the FBAM estimated frequency bands achieves the maximum accuracy relative to all models (see Figure 1 in the Supporting Information), further suggesting that FBAM estimates the frequency bands that best preserve the dynamics and differences in a population of interest.

## 7 Discussion

FBAM offers a new approach for the frequency band analysis of multiple stationary time series with extra spectral variability in a way that best preserves this variability in the resulting collapsed measures of power. It can identify distinct subpopulations in the frequency domain and includes selection criteria for selecting the number of subpopulations and frequency bands. A potentially useful extension is to allow for differing numbers of frequency bands across the subpopulations estimated by FBAM, which could be enabled by a properly designed varying‐length GA and penalization on the objective function.

FBAM has broad applicability to any collection of stationary signals that may exhibit extraspectral variability. For example, FBAM could be applied to understand the relationship between heart rate variability and stress [47] or the association between autonomic nervous impairment and hypertension [48]. FBAM could also be applied to electroencephalography series to evaluate the clinical efficacy of electroconvulsive therapy as a treatment for major depression [49] or to aid in the diagnosis of ADHD using pupil diameter time series [3]. Further, FBAM could be applied to center‐of‐pressure trajectories to assess the link between postural instability and fear of falling in patients with PD [50]. Lastly, our method is not constrained to the analysis of biomedical signals. For example, our method could be applied to a collection of seismic signals to potentially distinguish landslides from earthquakes and background noise in the frequency domain [51].

The primary objective of FBAM is to obtain summaries of a collection of power spectra as opposed to direct estimation of the underlying spectral densities. FBAM serves as an intermediary for deriving frequency band summary measures and is compatible with any consistent spectral estimator; although the current study utilizes multi‐taper estimates, consistent estimation of the loss function and selection criteria can also be achieved using other consistent spectral estimators. Further, FBAM uses a piecewise constant approximation to segment replicates and obtain summary measures. A consequence of this is that underlying spectral densities within a population are disentangled by FBAM on the basis of their discontinuous approximation rather than the full, continuous spectrum.

In this article, we make the assumption that the number of frequency bands required to adequately capture within‐subpopulation spectral variability does not vary across different subpopulations. This assumption can be easily relaxed. The loss function presented in Section 3 can easily accommodate sets of frequency bands that differ in the number of bands they define. However, the GA we have developed here would need to be modified to allow efficient exploration of a much more complex search space. One direction would be to implement a variable‐length GA with mutation operators that modify the number of frequency bands associated with any subpopulation. A modified optimization routine could then employ a two‐step procedure. The selection criterion for the number of frequency bands would be used first to select the number of bands in each subpopulation across solutions with varying numbers of subpopulations, followed by application of the selection criterion for the number of subpopulations to identify a final solution.

Though initially designed for stationary time series, FBAM could also be extended to nonstationary series with time‐varying spectra, allowing for time‐varying frequency bands. Another interesting future direction would be to generalize the FBAM framework to the problem of modeling a collection of long‐range dependent (LRD) time series, a prototype of which is provided in the Supporting Information. Theoretical investigations into the effect of increasing the number of time series would be another interesting direction of future research. Lastly, this framework could be extended to consider frequency band estimation for replicated multivariate time series.

## Funding

This work was supported by the National Institutes of Health (Grant No. R01GM140476) and the National Science Foundation (Grant No. CDS&E‐MSS‐2152950). The content is solely the responsibility of the authors and does not necessarily represent the official views of the National Institutes of Health or the National Science Foundation.

## Conflicts of Interest

The authors declare no conflicts of interest.

## Supporting information



Supporting Information is available online. It includes a detailed proof of all theorems, details of the GA, further details on the simulation studies in Section 5 and application in Section 6, sensitivity analyses for the number of tapers and weighting of selection criteria, and preliminary results for adapting the method for modeling LRD time series. Code written in R which implements the GA and optimization routine described in Section 4 and allows for replication of all of our simulation studies and gait variability analysis is available at https://github.com/brubakerconnor/fbam. Lastly, a Docker image which provides a virtual environment for reproducing all results in this article is available at https://hub.docker.com/r/brubakerconnor/fbam‐docker.

## Data Availability

The data that support the findings of this study are openly available in the Gait in Neurodegenerative Disease Database at https://doi.org/10.13026/C27G6C.
