# Supplementary material for: Frequency Band Analysis of Multiple Stationary Time Series
Source: Stat Med. 2026 Feb 12;45(3-5):e70412. doi: 10.1002/sim.70412 (PMC12899379; doi:10.1002/sim.70412)
Supplement: Supplementary file 1 — Supporting Information is available online. It includes a detailed proof of all theorems, details of the GA, further details on the simulation studies in Section 5 and application in Section 6, sensitivity analyses for the number of tapers and weighting of selection criteria, and preliminary results for adapting the method for modeling LRD time series. Code written in R which implements the GA and optimization routine described in Section 4 and allows for replication of all of our simulation studies and gait variability analysis is available at https://github.com/brubakerconnor/fbam. Lastly, a Docker image which provides a virtual environment for reproducing all results in this article is available at https://hub.docker.com/r/brubakerconnor/fbam‐docker. [file SIM-45-0-s001.pdf]

Supplementary Materials for *Frequency Band Analysis of Multiple Stationary Time Series*

Connor K. Brubaker[1], Jack Manning[2], Jennifer M. Yentes[2], and Scott A. Bruce[1]

[1] *Department of Statistics , Texas A&M University , College Station, Texas*
[2] *Department of Health & Kinesiology , Texas A&M University , College Station, Texas*

Contents

1 Proofs **1**

2 Details of the Genetic Algorithm **8**

3 Choosing the Number of Tapers **10**

4 Unequal Weighting of Selection Criteria **12**

5 Alternative Method for Frequency Band Estimation **12**

6 Logistic Regression Analysis of LF Summary Measures **14**

7 Modeling Long Range Dependence **15**

This document contains the supplementary material for the article "Frequency Band Analysis for Multiple Stationary Time Series." Section 1 presents proofs of theoretical results in the article. Section 2 provides details of our genetic algorithm for optimization of the FBAM objective function. Section 5 details the comparison study with an alternative method for simultaneous subpopulation and frequency band estimation. Section 6 details the comparison of different possible cutpoints for the low frequency band and its impact on classifying Huntington's disease patients. Section 7 describes a prototype and preliminary results for an extension of this work to characterize long range dependence in a collection of stationary time series.

1 Proofs

For convenience, we re-state some of the definitions in the article below. We then present some required preliminary results before proving the results presented in the article. In what follows, let $\{X_{jkt}\}$ for $j = 1, \ldots, J$ and $k = 1, \ldots, K_j$ be a set of observed time series of length T characterized by the stochastic transfer function model with the subject-level partition defined by $\mathbf{C}$. Also, denote by $g_{jk}(\omega)$ the underlying power spectrum of the kth replicate in subpopulation j and $\hat{g}_{jk}(\omega_m)$ the corresponding multitaper estimator of g_{jk} at Fourier frequency $\omega_m = 2\pi m/T$ for $m = 1, \ldots, \lfloor T/2 \rfloor - 1$ using R sine tapers. To ensure consistent estimation of the underlying spectra, we assume $R/T \to 0$ as $T \to \infty$ and $R \to \infty$ (Walden, 2000).

Definition 1 (Frequency Bands)**.** A **set of** L **frequency bands associated to subpopulation** j is the partition $\mathbf{W}_j = \{W_{j1}, \ldots, W_{jL}\}$ where $W_{jl} = [\omega^*_{j(l-1)}, \omega^*_{jl})$ for $l = 1, \ldots, L$ with $\omega^*_{j0} = 0$, $\omega^*_{jL} = 1/2$ and $0 < \omega^*_{j1} < \cdots < \omega^*_{j(L-1)} < 1/2$.

Definition 2 (Summary Measures of Power)**.** Conditional on sets of frequency bands $\mathbf{W}_1, \ldots, \mathbf{W}_J$ to each of the J subpopulations defined by a subject-level partition $\mathbf{C}$, the **replicate-specific collapsed measures of power** for the kth replicate in the jth subpopulation are defined as

$$y_{jkl} := \frac{1}{|W_{jl}|} \int_{W_{jl}} g_{jk}(\omega)\ d\omega \tag{1}$$

for $l = 1, \ldots, L$ where $|W_{jl}| := \omega^*_{jl} - \omega^*_{j(l-1)}$. The **average summary measures of power in subpopulation j** are

$$y_{j \cdot l} := \frac{1}{K_j} \sum_{k=1}^{K_j} y_{jkl} \tag{2}$$

where K_j is the number of replicates belonging to subpopulation j.

In the article, we present the following estimators of these quantities. Replicate-specific summary measures (1) are estimated with

$$\hat{y}_{jkl} = \frac{1}{M_{jl}} \sum_{\omega_m \in W_{jl}} \hat{g}_{jk}(\omega_m) \tag{3}$$

where M_{jl} is the number of Fourier frequencies ω_m contained within the frequency band W_{jl}. Average summary measures (2) are estimated with

$$\hat{y}_{j \cdot l} = \frac{1}{K_j} \sum_{k=1}^{K_j} \hat{y}_{jkl}. \tag{4}$$

Lemma 1. *For any subject-level partition* $\mathbf{C}$ *and associated sets of frequency bands* $\mathbf{W}_1, \ldots, \mathbf{W}_J$*,* $\hat{y}_{jkl} = y_{jkl} + \mathcal{O}_p(R/T) + O(T^{-1})$.

Proof. There are two sources of error in estimating y_{jkl} with $\hat{y}_{jkl}$, namely, the stochastic estimation error introduced by the multitaper estimator and the (non-stochastic) approximation error introduced by discretization of the integral in the definition of y_{jkl}. Consequently, we can decompose

$$\hat{y}_{jkl} - y_{jkl} = (\hat{y}_{jkl} - \tilde{y}_{jkl}) + (\tilde{y}_{jkl} - y_{jkl}) \tag{5}$$

where the first term is the random estimation error and the second term is the approximation error and where

$$\tilde{y}_{jkl} = \frac{1}{M_{jl}} \sum_{\omega_m \in W_{jl}} g_{jk}(\omega_m) \tag{6}$$

which is the same as (3) but using the true value of the underlying spectrum $g_{jk}(\omega_m)$ evaluated at the mth Fourier frequency ω_m. We will begin with examination of the first term.

Following asymptotic results for multitaper estimation of a stationary time series in Thomson (1982) and Percival and Walden (2020), under the mixing condition of Assumption 1 of the article, we have that

$$\hat{g}_{jk}(\omega) = g_{jk}(\omega) + \mathcal{O}_p(R/T). \tag{7}$$

The asymptotic term $\mathcal{O}_p(R/T)$ ensures that the length of the observed time series T grows faster than the number of tapers used in constructing the multitaper estimator R. This term also ensures that the asymptotic distribution of the multitaper approximation to the power spectrum holds. Consequently, the estimation error is $\hat{y}_{jkl} - \tilde{y}_{jkl} = \mathcal{O}_p(R/T)$.

To evaluate the second source of error in (5), we begin by defining $\underline{\omega}_{jl} := \min_{\omega_m \in W_{jl}} \omega_m$ and $\overline{\omega}_{jl} := \max_{\omega_m \in W_{jl}} \omega_m$ which are the minimum and maximum Fourier frequencies contained within the frequency band W_{jl}, respectively. First note that $\tilde{y}_{jkl}$ is a left-endpoint Riemann sum over the interval $[\underline{\omega}_{jl}, \overline{\omega}_{jl} + T^{-1}]$ with M_{jl} subintervals of length T^{-1}. Therefore, $M_{jl} = T(\overline{\omega}_{jl} + T^{-1} - \underline{\omega}_{jl})$. Write

$$\begin{aligned}
\tilde{y}_{jkl} - y_{jkl} &= \frac{1}{M_{jl}} \sum_{\omega_m \in W_{jl}} g_{jk}(\omega_m) - \frac{1}{|W_{jl}|} \int_{W_{jl}} g_{jk}(\omega)\, d\omega \\
&= \left(\frac{1}{\overline{\omega}_{jl} + T^{-1} - \underline{\omega}_{jl}} \sum_{\omega_m \in W_{jl}} g_{jk}(\omega_m) \frac{1}{T} - \frac{1}{\overline{\omega}_{jl} + T^{-1} - \underline{\omega}_{jl}} \int_{\underline{\omega}_{jl}}^{\overline{\omega}_{jl} + T^{-1}} g_{jk}(\omega)\, d\omega \right) \\
&+ \left(\frac{1}{\overline{\omega}_{jl} + T^{-1} - \underline{\omega}_{jl}} \int_{\underline{\omega}_{jl}}^{\overline{\omega}_{jl} + T^{-1}} g_{jk}(\omega)\, d\omega - \frac{1}{|W_{jl}|} \int_{W_{jl}} g_{jk}(\omega)\, d\omega \right)
\end{aligned}$$

Under Assumption 2 in the article, well-known results in numerical integration (e.g., Baker (1968)) tells us that the first difference on the last line above is $\mathcal{O}(T^{-1})$. The second term is a difference of integrals that intuitively becomes negligible as $T \to \infty$. To show this, write the first integral as

$$\frac{1}{\overline{\omega}_{jl} + T^{-1} - \underline{\omega}_{jl}} \left[\int_{\underline{\omega}_{jl}}^{\overline{\omega}_{jl}} g_{jk}(\omega)\, d\omega + \int_{\overline{\omega}_{jl}}^{\omega_{jl}^*} g_{jk}(\omega)\, d\omega + \int_{\omega_{jl}^*}^{\overline{\omega}_{jl} + T^{-1}} g_{jk}(\omega)\, d\omega \right]$$

and the second integral as

$$\frac{1}{|W_{jl}|} \left[\int_{\omega_{j(l-1)}^*}^{\underline{\omega}_{jl}} g_{jk}(\omega)\, d\omega + \int_{\underline{\omega}_{jl}}^{\overline{\omega}_{jl}} g_{jk}(\omega)\, d\omega + \int_{\overline{\omega}_{jl}}^{\omega_{jl}^*} g_{jk}(\omega)\, d\omega \right].$$

Their difference simplifies to

$$\frac{1}{\overline{\omega}_{jl}+T^{-1}-\underline{\omega}_{jl}}\int_{\omega_{jl}^*}^{\overline{\omega}_{jl}+T^{-1}} g_{jk}(\omega)\ d\omega - \frac{1}{|W_{jl}|}\int_{\omega_{j(l-1)}^*}^{\underline{\omega}_{jl}} g_{jk}(\omega)\ d\omega$$
$$+\left(\frac{1}{\overline{\omega}_{jl}+T^{-1}-\underline{\omega}_{jl}} - \frac{1}{|W_{jl}|}\right)\left(\int_{\underline{\omega}_{jl}}^{\overline{\omega}_{jl}} g_{jk}(\omega)\ d\omega + \int_{\overline{\omega}_{jl}}^{\omega_{jl}^*} g_{jk}(\omega)\ d\omega\right)$$

For the first term, we have that

$$\frac{1}{\overline{\omega}_{jl}+T^{-1}-\underline{\omega}_{jl}}\int_{\omega_{jl}^*}^{\overline{\omega}_{jl}+T^{-1}} g_{jk}(\omega)\ d\omega \leq \frac{\overline{\omega}_{jl}+T^{-1}-\omega_{jl}^*}{\overline{\omega}_{jl}+T^{-1}-\underline{\omega}_{jl}} \sup_{\omega\in[\overline{\omega}_{jl}+T^{-1},\omega_{jl}^*)]} g_{jk}(\omega) = \mathcal{O}(T^{-1})$$

and similarly for the second term,

$$\frac{1}{|W_{jl}|}\int_{\omega_{j(l-1)}^*}^{\underline{\omega}_{jl}} g_{jk}(\omega)\ d\omega \leq \frac{\underline{\omega}_{jl}-\omega_{j(l-1)}^*}{|W_{jl}|} \sup_{\omega\in[\overline{\omega}_{jl}+T^{-1},\omega_{jl}^*)]} g_{jk}(\omega) = \mathcal{O}(T^{-1}).$$

It is easy to see by similar argument that

$$\int_{\underline{\omega}_{jl}}^{\overline{\omega}_{jl}} g_{jk}(\omega)\ d\omega + \int_{\overline{\omega}_{jl}}^{\omega_{jl}^*} g_{jk}(\omega)\ d\omega = \mathcal{O}(1).$$

Finally, it can be shown that

$$\frac{1}{\overline{\omega}_{jl}+T^{-1}-\underline{\omega}_{jl}} - \frac{1}{|W_{jl}|} = \mathcal{O}(T^{-1})$$

Putting this all together, we have shown the approximation error is $\tilde{y}_{jkl} - y_{jkl} = \mathcal{O}(T^{-1})$ which gives the desired result. □

Lemma 2. *For any subject-level partition* $\mathbf{C}$ *and associated sets of frequency bands* $\mathbf{W}_1,\ldots,\mathbf{W}_J$, $\hat{y}_{j\cdot l} = y_{j\cdot l} + \mathcal{O}_p(R/T) + \mathcal{O}(T^{-1})$.

Proof. Substitution of the result of Lemma 1 into the definition of $\hat{y}_{j\cdot l}$ yields the result. □

Lemma 3. *For any subject-level partition* $\mathbf{C}$ *and associated sets of frequency bands* $\mathbf{W}_1,\ldots,\mathbf{W}_J$,

$$\frac{1}{T}\sum_{k=1}^{K_j}\sum_{\omega_m\in W_{jl}} (\hat{g}_{jk}(\omega_m) - \hat{y}_{j\cdot l})^2 - \left(\int_{W_{jl}} (g_{jk}(\omega) - y_{j\cdot l})^2\ d\omega\right) = \mathcal{O}_p(R/T) + \mathcal{O}(T^{-1}).$$

for each $j = 1,\ldots,J$ *and* $l = 1,\ldots,L$.

Proof. We begin by expanding the summands and integrand and comparing similar terms:

$$
\begin{aligned}
&\frac{1}{T}\sum_{k=1}^{K_j}\sum_{\omega_m\in W_{jl}} \left(\hat{g}_{jk}^2(\omega_m) - 2\hat{y}_{j\cdot l}\hat{g}_{jk}(\omega_m) + \hat{y}_{j\cdot l}^2\right) - \left(\int_{W_{jl}} g_{jk}^2(\omega) - 2y_{j\cdot l}g_{jk}(\omega) + y_{j\cdot l}^2 \; d\omega\right) \\
&\qquad = \frac{1}{T}\sum_{k=1}^{K_j}\sum_{\omega_m\in W_{jl}} \hat{g}_{jk}^2(\omega_m) - \int_{W_{jl}} g_{jk}^2(\omega)\; d\omega \\
&\qquad + 2y_{j\cdot l}\int_{W_{jl}} g_{jk}(\omega)\; d\omega - \frac{2\hat{y}_{j\cdot l}}{T}\sum_{k=1}^{K_j}\sum_{\omega_m\in W_{jl}} \hat{g}_{jk}(\omega_m) \\
&\qquad + \frac{1}{T}\sum_{k=1}^{K_j}\sum_{\omega_m\in W_{jl}} \hat{y}_{j\cdot l}^2 - \int_{W_{jl}} y_{j\cdot l}^2\; d\omega
\end{aligned}
$$

Application of (7) and an argument identical to that used in the proof of Lemma 1 tells us that

$$
\frac{1}{T}\sum_{k=1}^{K_j}\sum_{\omega_m\in W_{jl}} \hat{g}_{jk}^2(\omega_m) - \int_{W_{jl}} g_{jk}^2(\omega)\; d\omega = \mathcal{O}_p(R/T) + \mathcal{O}(T^{-1}).
$$

and

$$
2y_{j\cdot l}\int_{W_{jl}} g_{jk}(\omega)\; d\omega - \frac{2\hat{y}_{j\cdot l}}{T}\sum_{k=1}^{K_j}\sum_{\omega_m\in W_{jl}} \hat{g}_{jk}(\omega_m) = \mathcal{O}_p(R/T) + \mathcal{O}(T^{-1}).
$$

Finally, as a consequence of Lemma 1,

$$
\frac{1}{T}\sum_{k=1}^{K_j}\sum_{\omega_m\in W_{jl}} \hat{y}_{j\cdot l}^2 - \int_{W_{jl}} y_{j\cdot l}^2\; d\omega = \mathcal{O}_p(R/T) + \mathcal{O}(T^{-1}).
$$

Combining these together yields the result. □

We now re-state Theorems 1 and 2 from the article and present their proofs below.

Theorem 1. *Let* $\mathbf{C}$ *be a partition of* $\{X_{jk1}, \ldots, X_{jkT}\}$ *time series observations of length* T *into* $j = 1, \ldots, J$ *subpopulations with* $k = 1, \ldots, K_j$ *subjects each whose underlying spectra are characterized by the stochastic transfer function model with a possibly unknown number of subpopulations. Let* $\mathbf{W}_1, \ldots, \mathbf{W}_J$ *be sets of* L *frequency bands associated to each of the subpopulations. Then*

$$
\hat{\mathcal{L}}(\mathbf{C}, \mathbf{W}_1, \ldots, \mathbf{W}_J) = \mathcal{L}(\mathbf{C}, \mathbf{W}_1, \ldots, \mathbf{W}_J) + \mathcal{O}_P\left(\frac{R}{T}\right) + \mathcal{O}\left(\frac{1}{T}\right). \tag{8}
$$

Proof. The proof for Theorem 1 is analogous to that of Lemma 1. Define

$$
\tilde{\mathcal{L}}(\mathbf{C}, \mathbf{W}_1, \ldots, \mathbf{W}_J) = \frac{1}{T}\sum_{j=1}^{J}\sum_{k=1}^{K_j}\sum_{l=1}^{L}\sum_{\omega\in W_{jl}} (g_{jk}(\omega) - \tilde{y}_{j\cdot l})^2.
$$

where $\tilde{y}_{j\cdot l} = K_j^{-1} \sum_{k=1}^{K_j} \tilde{y}_{jkl}$ and $\tilde{y}_{jkl}$ is defined in (6). It is easy to show that $\hat{\mathcal{L}}(\mathbf{C}, \mathbf{W}_1, \ldots, \mathbf{W}_J) = \tilde{\mathcal{L}}(\mathbf{C}, \mathbf{W}_1, \ldots, \mathbf{W}_J) + \mathcal{O}_p(R/T)$. Invoking Lemma 3, we arrive at the desired result. □

Theorem 2. *As before, let* $\mathbf{C}$ *be a partition of subjects into* J *subpopulations and* $\mathbf{W}_1, \ldots, \mathbf{W}_J$ *be sets of* L *frequency bands associated to each of the subpopulations. Assume that* $\sum_{k=1}^{K_j} \|(g_{jk}(\omega) - \mu_{j\cdot l})\mathbf{1}_{W_{jl} \cup W_{j,l+1}}(\omega)\|_2^2 = c_{jl}$ *and* $\|y_i(\omega) - y_j(\omega)\|_2^2 = d_{ij}$ *are bounded away from 0 such that* $c_{jl} \geq \epsilon$ *for* $j = 1, \ldots, J$ *and* $l = 1, \ldots, L-1$ *and* $d_{ij} \geq \epsilon$ *for each* $i \neq j$ *for some fixed constant* $\epsilon > 0$*. Then* $\hat{R}_{jl}^{(1)} = R_{jl}^{(1)} + \mathcal{O}_P(R/T) + \mathcal{O}(T^{-1})$ *and* $\hat{R}_{ij}^{(2)} = R_{ij}^{(2)} + \mathcal{O}_P(R/T) + \mathcal{O}(T^{-1})$*.*

Proof. We begin by analyzing $\hat{R}_l^{(1)}$ first. Write

$$\hat{R}_{jl}^{(1)} = \frac{\left(\frac{1}{T}\sum_{k=1}^{K_j}\sum_{\omega \in W_{jl}}(\hat{g}_{jk}(\omega) - \hat{y}_{j\cdot l})^2\right)^{1/2} + \left(\frac{1}{T}\sum_{k=1}^{K_j}\sum_{\omega \in W_{j,l+1}}(\hat{g}_{jk}(\omega) - \hat{y}_{j\cdot(l+1)})^2\right)^{1/2}}{\left(\frac{1}{T}\sum_{k=1}^{K_j}\sum_{\omega \in W_{jl} \cup W_{j,l+1}}(\hat{g}_{jk}(\omega) - \hat{\mu}_{jl})^2\right)^{1/2}}$$

where the scaling factor T^{-1} has been added but does not change the value of $\hat{R}_{jl}^{(1)}$. Applying Lemma 3 to each term in the numerator and to the denominator and using Taylor series expansions, we have that

$$\hat{R}_{jl}^{(1)} = R_{jl}^{(1)} + \mathcal{O}_P(R/T) + \mathcal{O}(T^{-1}).$$

A similar argument yields that second result. □

Lemma 4. *Let* $\{X_{1t}\}, \ldots, \{X_{nt}\}$ *be a set of* $i = 1, \ldots, n$ *sequences of random variables such that* $X_{it} = \theta + \mathcal{O}_P(a_t) + \mathcal{O}(b_t)$*. Then, for a fixed* t*,* $\max_i X_{it} = \theta + \mathcal{O}_P(a_t) + \mathcal{O}(b_t)$*.*

Proof. Fix t and write $X_{it} = \theta + Y_{it} + Z_{it}$ where $Y_{it} = \mathcal{O}_P(a_t)$ and $Z_{it} = \mathcal{O}(b_t)$ for each $i = 1, \ldots, n$. By definition, for each i and any $\varepsilon > 0$, there exists a constant $M_i > 0$ and integer T_i such that $P(|Y_{it}| > M_i a_t) < \varepsilon/n$ for $t > T_i$. If we take $M = \max_i M_i$ and $T = \max_i T_i$, then clearly $P(|Y_{it}| > M a_n) < \varepsilon/n$ for $t > T$ and all i. Now,

$$P(\max_i |Y_{it}| > M a_n) = P\left(\bigcup_{i=1}^{n}\{|Y_{it}| > M a_n\}\right) \leq \sum_{i=1}^{n} P(|Y_{it}| > M a_n) < \sum_{i=1}^{n} \varepsilon/n = \varepsilon$$

for each i and $t > T$. Therefore, $max_i Y_{it} = \mathcal{O}_P(a_n)$. A similar line of argument shows that $\max_i Z_i = \mathcal{O}(b_n)$. Combining these leads to the desired result. □

Corollary 1. *Consider the same settings and assumptions as Theorem 2. Then*

$$\hat{S}^{(1)}(\mathbf{C}, \mathbf{W}_1, \ldots, \mathbf{W}_J \mid J, L) = S^{(1)}(\mathbf{C}, \mathbf{W}_1, \ldots, \mathbf{W}_J \mid J, L) + \mathcal{O}_P(R/T) + \mathcal{O}(T^{-1})$$

and

$$\hat{S}^{(2)}(\mathbf{C}, \mathbf{W}_1, \ldots, \mathbf{W}_J \mid J, L) = S^{(2)}(\mathbf{C}, \mathbf{W}_1, \ldots, \mathbf{W}_J \mid J, L) + \mathcal{O}_P(R/T) + \mathcal{O}(T^{-1}).$$

Proof. By definition,

$$\begin{aligned}
\hat{S}^{(1)}(\mathbf{C}, \mathbf{W}_1, \ldots, \mathbf{W}_J \mid J, L) &= (JL)^{-1} \sum_{j=1}^{J} \sum_{l=1}^{L-1} \hat{R}_{jl}^{(1)}(\mathbf{C}, \mathbf{W}_1, \ldots, \mathbf{W}_J \mid J, L) \\
&= (JL)^{-1} \sum_{j=1}^{J} \sum_{l=1}^{L-1} R_{jl}^{(1)}(\mathbf{C}, \mathbf{W}_1, \ldots, \mathbf{W}_J \mid J, L) + \mathcal{O}_P(R/T) + \mathcal{O}(T^{-1}) \\
&= S^{(1)}(\mathbf{C}, \mathbf{W}_1, \ldots, \mathbf{W}_J \mid J, L) + \mathcal{O}_P(R/T) + \mathcal{O}(T^{-1}).
\end{aligned}$$

A similar derivation utilizing the result of Lemma 4 yields the second result. □

Theorem 3. *Consider the same settings and assumptions as Theorems 1 and 2. Let* $\mathbf{J}$ *and* $\mathbf{L}$ *be finite sets of parameter values for the number of subpopulations and number of frequency bands under consideration, respectively, such that* $\min(\mathbf{J}) \geq 2$ *and* $\min(\mathbf{L}) \geq 2$*. For convenience, write* $\theta_{(J,L)} \equiv (\mathbf{C}, \mathbf{W}_1, \ldots, \mathbf{W}_J)$ *where* $\mathbf{C}$ *is a partition of observed time series into* J *subpopulations and* $\mathbf{W}_j$ *is a set of* L *frequency bands associated to the* j*th subpopulation. Define the functions* $n_J(\theta_{(J,L)}) = J$ *and* $n_L(\theta_{(J,L)}) = L$*. Let* $\mathcal{G} = \{\hat{\theta}_{(J,L)} \mid (J, L) \in \mathbf{J} \times \mathbf{L}\}$ *where* $\hat{\theta}_{(J,L)} = \arg\min \mathcal{L}(\theta \mid J, L)$*. Lastly, define* $\hat{J} = n_J(\arg\min_{\theta \in \mathcal{G}} \hat{S}(\theta))$*,* $\hat{L} = n_L(\arg\min_{\theta \in \mathcal{G}} \hat{S}(\theta))$*,* $J^* = n_J(\arg\min_{\theta \in \mathcal{G}} S(\theta))$*, and* $L^* = n_L(\arg\min_{\theta \in \mathcal{G}} S(\theta))$*. Assume that* $S(\theta_{(J^*,L^*)}) < S(\theta)$ *for all* $\theta \in \mathcal{G}$*. Then* $\hat{J} \to_P J^*$ *and* $\hat{L} \to_P L^*$*.*

Proof. By definition of $\hat{J}$ and $\hat{L}$, we have that $\hat{S}(\theta_{(\hat{J},\hat{L})}) \leq \hat{S}(\theta_{(J^*,L^*)})$. Consequently,

$$\begin{aligned}
S(\theta_{(\hat{J},\hat{L})}) &= S(\theta_{(J^*,L^*)}) + S(\theta_{(\hat{J},\hat{L})}) - \hat{S}(\theta_{(\hat{J},\hat{L})}) + \hat{S}(\theta_{(\hat{J},\hat{L})}) - S(\theta_{(J^*,L^*)}) \\
&\leq S(\theta_{(J^*,L^*)}) + S(\theta_{(\hat{J},\hat{L})}) - \hat{S}(\theta_{(\hat{J},\hat{L})}) + \hat{S}(\theta_{(J^*,L^*)}) - S(\theta_{(J^*,L^*)}) \\
&\leq S(\theta_{(J^*,L^*)}) + 2 \sup_{\theta \in \mathcal{G}} |\hat{S}(\theta) - S(\theta)|.
\end{aligned}$$

But by definition of J^* and L^*, we have that $S(\theta_{(J^*,L^*)}) \leq \hat{S}(\theta_{(\hat{J},\hat{L})})$ so

$$S(\theta_{(J^*,L^*)}) \leq S(\theta_{(\hat{J},\hat{L})}) \leq S(\theta_{(J^*,L^*)}) + 2 \sup_{\theta \in \mathcal{G}} |\hat{S}(\theta) - S(\theta)|.$$

By Corollary 1, since $\mathcal{G}$ is a finite set, $\sup_{\theta \in \mathcal{G}} |\hat{S}(\theta) - S(\theta)| = \mathcal{O}_P(R/T) + \mathcal{O}(T^{-1}) = \mathcal{O}_P(R/T)$ where the deterministic term has been absorbed by the stochastic one. The above assumption that $R/T \to 0$ as $T \to \infty$ implies that $\sup_{\theta \in \mathcal{G}} |\hat{S}(\theta) - S(\theta)| = o_P(1)$ which yields the desired result. □

2 Details of the Genetic Algorithm

This section walks through the details of our implementation of an genetic algorithm for optimization of the FBAM objective function.

Chromosome representation. There are two options for encoding candidate solutions. The first is a label-based encoding where the integer label of each replicate in the data along with the frequency bands associated to each (non-empty) subpopulation is encoded by any candidate solution. The second is a center-based encoding where the frequency bands associated to each subpopulation as well as a subpopulation-specific set of associated average summary measures are encoded instead. Replicates are then assigned a subpopulation on the basis of minimum L^2 distance to obtain a partition $\mathbf{C}$. We will use a center-based encoding a label-based encoding can lead to significantly longer convergence times even for moderately sized data sets Bandyopadhyay (2011).

Let J be the number of subpopulations and L the number of frequency bands associated to each cluster. The pth chromosome is written $\boldsymbol{\delta}_p = (\boldsymbol{m}_1, \dots, \boldsymbol{m}_J; \boldsymbol{z}_1, \dots, \boldsymbol{z}_J)$ for $p = 1, \dots, P$ user specified number of chromosomes (population size). In this representation, $\boldsymbol{m}_j = (m_{j1}, \dots, m_{jB-1})^\top$ where m_{jb} is the index of the m_{jl}th Fourier frequency that serves as the boundary between the lth and $(l+1)$th frequency band associated to the jth subpopulation. Also, $\boldsymbol{z}_j = (z_{j\cdot 1}, \dots, z_{j\cdot L})^\top$ where $z_{j\cdot l}$ is the average summary measure of power on the lth band for the jth subpopulation. In the succeeding discussion, we define $m_{j0} = 0$ and $m_{jB} = M + 1$ for each j. These are not included in the chromosomal representation since they are fixed for each j and all p.

Population initialization. Each of the P chromosomes is initialized in two steps using a data-guided approach as described in the subsequent paragraphs. Empirical observation suggested that using the data to guide initialization led to faster convergence times and lower likelihood of convergence to sub-optimal solutions. Further, we default to using a population size of $P = 50$ based on empirical experimentation and the common wisdom of using small to moderate population sizes (Eiben and Smith, 2015).

The frequency band boundaries associated to each subpopulation by all chromosomes are initialized by finding a set of equally spaced $L - 1$ integers between 2 and M, inclusive. The summary measures of each chromosome, $\boldsymbol{z}_j$ for $j = 1, \dots, J$, are initialized independently of each other by randomly sampling one replicate-specific spectrum $\hat{g}_k$ from the collection of K total replicates in the input data. Then for $l = 1, \dots, L$, we initialize

$$z_{j\cdot l} = \frac{1}{m_{jl} - m_{jl-1}} \sum_{m=m_{jl-1}}^{m_{jl}-1} \hat{g}_k(\omega_m).$$

Loss computation. Each replicate of the input data is assigned to the jth subpopulation via the minimum L^2 distance rule. We assign replicates to the jth subpopulation such that

$$\sum_{l=1}^{L} \sum_{m=m_{jb-1}}^{m_{jb}-1} (\hat{g}_k(\omega_m) - z_{j \cdot l})^2$$

is minimized. When $J = 1$, this step is skipped. Then we compute

$$\hat{y}_{j \cdot l} = \frac{1}{K_j(m_{jl} - m_{jl-1})} \sum_{k=1}^{K_j} \sum_{m=m_{jl-1}}^{m_{jl}-1} \hat{g}_{jk}(\omega_m)$$

where K_j is the number of replicates assigned to the jth subpopulation and $\hat{g}_{jk}$ is the kth replicate assigned to the jth subpopulation. The value of the loss function associated to the pth chromosome is then

$$\sum_{j=1}^{J} \sum_{k=1}^{K_j} \sum_{l=1}^{L} \sum_{m=m_{jl-1}}^{m_{jl}-1} (\hat{g}_k(\omega_m) - \hat{y}_{j \cdot l})^2$$

Note that the loss function is computed using the average collapsed measures computed directly from the data, not the values $z_{j \cdot 1}, \ldots, z_{j \cdot L}$ encoded by $\boldsymbol{\delta}_p$.

Infeasible solutions. The assignment rule above does not guarantee J non-empty subpopulations. We associate to any chromosome $\boldsymbol{\delta}_p$ that yields empty clusters a loss function value of ∞. In our practical implementation, we use set the fitness of an infeasible solution to `1e50`.

Selection and population management. Parent chromosomes are selected inversely proportional to the loss function to which they are associated (a smaller loss function means you are more likely to be selected). These chromosomes are subjected to the mutation operator described in the next paragraph. After mutation, all chromosomes not selected at this step are discarded.

Mutation. Though commonly employed in genetic algorithms, we omit a crossover operation since it was not observed to significantly improve the quality of solutions or algorithm run time. Each value of the chromosome is subject to change with probability p_m. The method by which mutation is performed differs between Fourier frequency boundaries m_{jl} and the encoded cluster-specific mean collapsed measures $z_{j \cdot l}$. To prevent the generation of infeasible solutions, we reject a mutation and preserve the original chromosome if the mutation results in an infeasible solution.

Mutation of band boundaries m_{jl}. Define $\Delta_{jl} = m_{jl} - m_{j(l-1)} - 1$ which is the number of Fourier frequencies minus one contained in the lth band associated to the jth subpopulation. A band boundary m_{jl} selected for mutation is modified by adding to it a value

$$\eta \sim \text{TN}(\mu = 0, \sigma^2 = \max_l(\Delta_{jl})/8, a = -\Delta_{j(l-1)}, b = \Delta_{jl})$$

where $[n]$ denotes the nearest integer to n and $\text{TN}(\mu, \sigma^2, a, b)$ denotes a truncated normal distribution with mean μ, variance σ^2, lower bound a, and upper bound b. Mutation in this manner eliminates the need for any user-specified parameters and results in more likely small changes while allowing for occasional large changes in m_{jl}. Additionally, this guarantees preservation of the ordering of the m_{jl}.

Mutation of average summary measures. An average summary measure $z_{j\cdot l}$ selected for mutation is modified by sampling a new value

$$z'_{j\cdot l} \sim \text{TN}(\mu = z_{j\cdot l}, \sigma^2 = Z/8, a = 0, b = Z)$$

where $Z := \sqrt{\log(\max_{k,\omega_m} \hat{g}_k(\omega_m))}$ is the maximum spectral estimate among all replicate-specific spectra and across all Fourier frequencies. Similar to before, this requires no user-specified parameters and ensures that the average summary measures stay within the range of their possible values.

Mutation Rate. We default to a mutation rate of $p_m = 0.15$.

Declaration of convergence. The algorithm is terminated after at least one of two convergence criteria is met. The first of these is after the Gth generation. The second of these is if the maximum fitness of the population does not significantly improve after G' generations where a "significant" improvement is defined as at least a 1% increase in maximum fitness from the previous generation. We default to using $G = 500$ and $G' = 100$.

3 Choosing the Number of Tapers

In this section, we demonstrate how the choice of the number of tapers R used in multitaper estimation affects performance of FBAM. Table 1 reports the adjusted Rand Index (ARI) and estimated number of subpopulations $\hat{J}$ and frequency bands $\hat{L}$ averaged over 100 independent replications for each model described in the article with $K_j = 20$ and $T = 1000$. We consider $R = \lfloor T^\alpha \rfloor$ for $\alpha = 0.2, 0.4, 0.5, 0.6$, and 0.8. Note

**Model**	α	**ARI**	$\hat{J}$	$\hat{L}$
Model 1	0.2	0.55 (0.02)	2.00 (0.00)	2.54 (0.70)
	0.4	0.75 (0.23)	2.45 (0.5)	2.77 (0.42)
	0.5	0.61 (0.17)	2.16 (0.37)	2.10 (0.30)
	0.6	0.99 (0.06)	2.98 (0.14)	2.97 (0.17)
	0.8	0.99 (0.05)	2.99 (0.10)	2.94 (0.49)
Model 2(a)	0.2	0.33 (0.07)	2.09 (0.32)	3.12 (0.41)
	0.4	0.34 (0.07)	2.22 (0.42)	3.03 (0.17)
	0.5	0.35 (0.07)	2.40 (0.59)	3.00 (0.00)
	0.6	0.34 (0.08)	2.3 (0.56)	3.00 (0.00)
	0.8	0.28 (0.09)	3.58 (1.22)	3.04 (0.28)
Model 2(b)	0.2	0.57 (0.16)	2.31 (0.46)	3.09 (0.40)
	0.4	0.63 (0.17)	2.79 (0.69)	3.02 (0.20)
	0.5	0.65 (0.18)	2.97 (0.74)	3.00 (0.00)
	0.6	0.7 (0.17)	2.87 (0.56)	3.00 (0.00)
	0.8	0.57 (0.16)	2.81 (1.02)	3.00 (0.00)
Model 2(c)	0.2	0.85 (0.20)	2.68 (0.47)	3.02 (0.14)
	0.4	0.99 (0.03)	3.01 (0.10)	3.00 (0.00)
	0.5	0.98 (0.07)	3.00 (0.20)	3.00 (0.00)
	0.6	0.99 (0.02)	3.00 (0.00)	3.00 (0.00)
	0.8	0.9 (0.16)	2.91 (0.55)	3.01 (0.1)
Model 3	0.2	0.62 (0.15)	2.15 (0.36)	4.47 (0.74)
	0.4	0.85 (0.20)	2.68 (0.47)	4.09 (0.29)
	0.5	0.91 (0.17)	2.81 (0.39)	4.00 (0.00)
	0.6	0.85 (0.20)	2.67 (0.47)	4.04 (0.20)
	0.8	0.53 (0.06)	2.01 (0.10)	2.97 (0.44)

Table 1: Mean (SD) of Adjusted Rand Index, selected number of subpopulations $\hat{J}$, and selected number of frequency bands $\hat{L}$ for 100 independent replications of each model where the spectra have been estimated using $R = \lfloor T^{\alpha} \rfloor$ sine tapers.

that $\alpha = 0.5$ corresponds to the number of tapers used in the main simulation study. We reproduce values from Table 1 of the main article for $\alpha = 0.5$ in Table 1.

For Model 1, using more tapers than those corresponding to $\alpha = 0.5$ yields better subpopulation segmentation reflected by a higher ARI and values for $\hat{J}$ and $\hat{L}$ closer to the "true" value of 3 than when $\alpha = 0.5$. However, for the rest of the models, we see a degradation in performance when the number of tapers is set too high. This can be explained by the shape of the underlying spectra in Model 1 versus the other models. The underlying spectra in Model 1 closely resemble piecewise constant functions, so additional tapers help recover this smoother structure. In the case of the other models, the spectra are more dynamic, so including too many tapers will oversmooth the estimates of the underlying spectra, reducing FBAM's ability to discern between subpopulations. Additionally, the smoothing masks the dynamic shape of the underlying spectra, leading FBAM to select fewer bands than what is necessary. In these models, we see that choosing $R = \lfloor T^{\alpha} \rfloor$ for $\alpha \in [0.4, 0.6]$ leads to the best performance.

4 Unequal Weighting of Selection Criteria

Write $\theta_{(J,L)} = (\mathbf{C}, \mathbf{W}_1, \ldots, \mathbf{W}_J)$ and define $\mathcal{G} = \{\hat{\theta}_{(J,L)} \mid (J, L) \in \mathbf{J} \times \mathbf{L}\}$ where $\hat{\theta}_{(J,L)} = \arg\min \hat{\mathcal{L}}(\theta \mid J, L)$. Set $a_1 = \sup_{\theta \in \mathcal{G}} \hat{S}^{(1)}(\theta \mid J, L)$ and $a_2 = \sup_{\theta \in \mathcal{G}} \hat{S}^{(2)}(\theta \mid J, L)$. Then the solution $\theta \in \mathcal{G}$ that minimizes $a_1^{-1}\hat{S}^{(1)}(\theta \mid J, L) + a_2^{-1}\hat{S}^{(2)}(\theta \mid J, L)$ is taken as the final solution. Scaling in this manner ensures that no one criterion influences the choice of solution more than the other since the choice of J and L are equally important. In this section, we investigate the trade off between these criterion by considering a joint selection criteria of the form

$$\alpha[a_1^{-1}\hat{S}^{(1)}(\theta \mid J, L)] + (1 - \alpha)[a_2^{-1}\hat{S}^{(2)}(\theta \mid J, L)]$$

for $\alpha \in (0, 1)$. Table 2 summarizes the effect of different choices of α on the mean ARI, choice of J and choice of L for each model when $K_j = 30$ and $T = 1000$. Across all models, increasing α leads to estimating fewer subpopulations. However, decreasing α leads to estimating fewer frequency bands only in Model 1. In the other models, the choice of L is insensitive to the choice of α. In general, an unequal weighting leads to less parsimonious solutions in either the choice of J or L whereas an equal weighting, and even a weighting with α near 0.5, leads to reasonable solutions across all models.

5 Alternative Method for Frequency Band Estimation

To the best of our knowledge, there are no existing methods that immediately offer frequency band and subpopulation estimation, so we have constructed an ad-hoc method based on functional clustering (Zhang

Model	α	ARI	J	L
Model 1	0.1	0.96 (0.08)	3.41 (0.75)	2.06 (0.24)
	0.2	0.99 (0.04)	3.11 (0.31)	2.34 (0.48)
	0.3	1.00 (0.02)	3.02 (0.14)	2.68 (0.47)
	0.4	1.00 (0.00)	3.00 (0.00)	2.89 (0.31)
	0.5	1.00 (0.00)	3.00 (0.00)	2.98 (0.14)
	0.6	1.00 (0.00)	3.00 (0.00)	2.99 (0.10)
	0.7	1.00 (0.04)	2.99 (0.10)	3.00 (0.00)
	0.8	1.00 (0.04)	2.99 (0.10)	3.00 (0.00)
	0.9	1.00 (0.04)	2.99 (0.10)	3.00 (0.00)
Model 2(a)	0.1	0.26 (0.05)	4.77 (0.84)	3.00 (0.00)
	0.2	0.26 (0.05)	4.65 (0.85)	3.00 (0.00)
	0.3	0.27 (0.05)	4.40 (0.89)	3.00 (0.00)
	0.4	0.28 (0.05)	4.16 (0.83)	3.00 (0.00)
	0.5	0.29 (0.05)	3.72 (0.70)	3.00 (0.00)
	0.6	0.30 (0.06)	3.45 (0.66)	3.00 (0.00)
	0.7	0.30 (0.06)	3.27 (0.58)	3.00 (0.00)
	0.8	0.32 (0.07)	2.87 (0.63)	3.00 (0.00)
	0.9	0.34 (0.07)	2.44 (0.54)	3.00 (0.00)
Model 2(b)	0.1	0.54 (0.08)	5.41 (0.68)	3.00 (0.00)
	0.2	0.55 (0.09)	5.29 (0.74)	3.00 (0.00)
	0.3	0.56 (0.09)	5.14 (0.78)	3.00 (0.00)
	0.4	0.57 (0.10)	4.92 (0.80)	3.00 (0.00)
	0.5	0.59 (0.10)	4.73 (0.79)	3.00 (0.00)
	0.6	0.62 (0.12)	4.32 (0.80)	3.00 (0.00)
	0.7	0.67 (0.13)	3.84 (0.73)	3.00 (0.00)
	0.8	0.74 (0.15)	3.33 (0.53)	3.00 (0.00)
	0.9	0.71 (0.18)	2.80 (0.53)	3.00 (0.00)
Model 2(c)	0.1	0.72 (0.11)	5.19 (0.79)	3.00 (0.00)
	0.2	0.73 (0.12)	5.09 (0.87)	3.00 (0.00)
	0.3	0.76 (0.13)	4.80 (0.98)	3.00 (0.00)
	0.4	0.83 (0.14)	4.25 (1.04)	3.00 (0.00)
	0.5	0.89 (0.13)	3.80 (0.97)	3.00 (0.00)
	0.6	0.95 (0.10)	3.37 (0.75)	3.00 (0.00)
	0.7	0.99 (0.04)	3.08 (0.31)	3.00 (0.00)
	0.8	0.99 (0.03)	3.03 (0.17)	3.00 (0.00)
	0.9	1.00 (0.01)	3.00 (0.00)	3.07 (0.38)
Model 3	0.1	0.93 (0.09)	3.59 (0.76)	4.00 (0.00)
	0.2	0.96 (0.07)	3.33 (0.59)	4.00 (0.00)
	0.3	0.98 (0.05)	3.18 (0.38)	4.00 (0.00)
	0.4	0.99 (0.03)	3.06 (0.24)	4.00 (0.00)
	0.5	1.00 (0.02)	3.02 (0.14)	4.00 (0.00)
	0.6	1.00 (0.01)	3.00 (0.00)	4.00 (0.00)
	0.7	0.99 (0.04)	2.99 (0.10)	4.00 (0.00)
	0.8	0.98 (0.08)	2.97 (0.17)	4.01 (0.10)
	0.9	0.92 (0.17)	2.82 (0.38)	4.14 (0.38)

Table 2: Mean (standard deviation) of the ARI, the number of subpopulations J and frequency bands L of the final solutions selected using a convex combination of the scaled selection criteria for various values of α when $K_j = 30$ and $T = 1000$.

and Parnell, 2023) and the Bayesian mixture auto-regressive decomposition (BMARD) (Granados-Garcia et al., 2022). BMARD is a methodology for the identification of frequency bands in brain electrical activity signals and models a replicate-specific spectrum as a mixture of second-order autoregressive spectra with mid-frequency peaks and associated bandwidths. After estimation with BMARD, additional post-processing steps are required to obtain a frequency partition.

$$\hat{g}_k(\omega_m) = \sum_{b=0}^{B} c_{kb} \cos\left(\frac{2\pi b \omega_m}{2M}\right)$$

where B is the number of basis functions and $\omega_m = m/T$ for $m \in \{-M, \ldots, M\} \setminus \{0\}$ where $M = \lfloor T/2 \rfloor - 1$. The resulting vectors $\mathbf{c}_k = (c_{k1}, \ldots, c_{kB})$ are then clustered using the J-means algorithm where J is the number of desired subpopulations specified *a priori* to obtain a subject-level partition $\mathbf{C}$.

BMARD is then applied to each replicate-specific periodogram to estimate the number of AR(2) components as well as their respective peaks and bandwidths. MCMC sampling for this step and their subsequent processing as described by the original authors is done entirely in parallel. We used their MCMC sampler (available at `https://github.com/Cuauhtemoctzin/BMARD`) to obtain 20000 samples across 3 chains, discarding the first $12,000$ as burn-in.

Within each subpopulation defined by $\mathbf{C}$, we determine the most common number of components estimated by BMARD across the replicates assigned to that subpopulation by the first step which we call η_j and may vary across the J subpopulations. Let $\hat{\psi}_{jkl}$ for $k = 1, \ldots, K_j$ and $l = 1, \ldots, \eta_j$ be the BMARD-estimated peak of the lth component of the kth replicate-specific spectrum in the jth subpopulation for $j = 1, \ldots, J$. For convenience, define $\psi_{jk0} = 0$ and $\psi_{jk(\eta_j+1)} = 1/2$. For each replicate in the jth subpopulation that was estimated to have η_j components by BMARD, we construct a set frequency bands $\mathbf{W}_{jk} = \{W_{jk1}, \ldots, W_{jk(\eta_j+1)}\}$ whose boundaries are defined by the midpoints between each replicate-specific peak, that is, $W_{jkl} = [(\hat{\psi}_{jk(l-1)} + \hat{\psi}_{jkl})/2, (\hat{\psi}_{jkl} + \hat{\psi}_{jk(l+1)})/2)$ for $l = 1, \ldots, \eta_j$. Finally, the frequency bands $\mathbf{W}_{jk}$ that minimize the FBAM objective function across all replicates within the jth subpopulation is chosen as the set of bands associated to that subpopulation.

6 Logistic Regression Analysis of LF Summary Measures

To study the informative capacity of the summary measures of power estimated by FBAM, we use them for predicting the presence of Huntington's disease (HD) versus everything else (Control, ALS, and PD). For this binary classification task, we employed logistic regression (LR). For comparison, a baseline LR model was fit using only age, height, and average gait speed. For each possible boundary defining the LF band, we

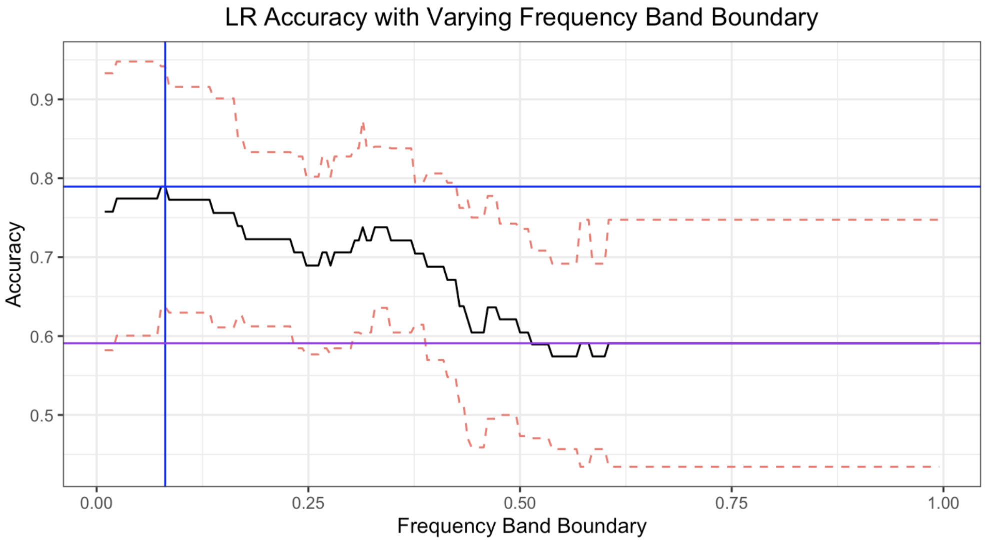

Figure 1: 6-fold cross validated accuracy (solid black line) plus or minus one standard error (dashed red lines) of the logistic regression model used to classify HD using LF summary measures of power as a function of the boundary that defines the LF frequency band. The vertical and horizontal blue lines mark the FBAM estimated frequency band and resulting cross-validated mean accuracy. The purple line marks the same accuracy of a baseline model fit only with age, height, and gait speed as covariates.

computed the associated replicate-specific LF summary measures and used these as features in a LR model along with age, height, and average gait speed. 6-fold cross validation was used to estimate the out-of-sample classification accuracy of each LR model. Figure 1 displays the cross-validated accuracy as a function of the LF frequency band boundary. It can be clearly seen here that the model which uses the FBAM estimated LF summary measures outperforms the baseline model and achieves the maximum accuracy relative to all models considered.

7 Modeling Long Range Dependence

Time series with long memory or long-range dependence (LRD) have spectra that exhibit a power law near frequency zero. Many of the estimated spectra observed in the gait variability study seem to exhibit this kind of behavior. However, the pre-processing of the stride interval series in this analysis includes a high pass filter that practically eliminates power at frequencies near zero associated with LRD. The visual resemblance to LRD in the estimated spectra is then likely due to increased low frequency power associated with the effect of turning strategy utilized by many subjects as they walk up and down a hallway to complete the experiment. A pivot turn strategy, used by some participants, leads to slightly longer time between steps during turns since participants are pivoting on one foot before taking the next step in the opposite direction. This results in increased power at lower frequencies associated with cycles of time that it takes a participant

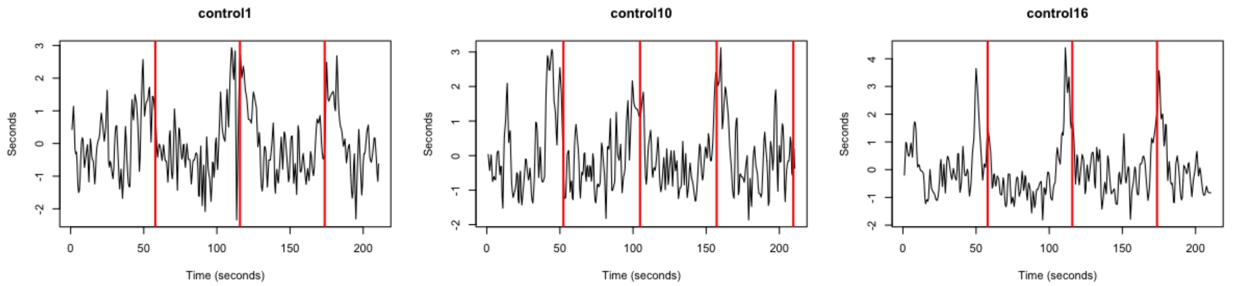

Figure 2: Stride interval time series for 3 healthy controls. The red lines mark the approximate locations in time where a turn was taken at the end of the hall.

to walk the length of the hall. An example of this can be seen in a few of the healthy controls as shown in Figure 2. The red vertical lines in these plots show the approximate times at which turns were taken at the end of the hall. These times were determined from knowledge of the hall length and reported average gait speed of that participant.

However, in the case that a collection of independent time series does exhibit LRD behavior, FBAM is readily extended to handle this. For a stationary, long-range dependent time series, the power spectrum is often modeled by $f(\omega) \sim C\omega^{-\alpha}$ for $C > 0$ and $0 < \alpha < 1$ or, equivalently, as $f(\omega) \sim C\omega^{1-2H}$ where H is the Hurst exponent. The Hurst exponent measures the degree of long-term memory or persistence in a time series, quantifying how future values are influenced by past behavior. A process with a Hurst exponent greater than 0.5 is characterized by long-term positive autocorrelation.

Now consider a collection of $k = 1, \ldots, K$ independent long-range dependent processes X_{kt} with replicate-specific power spectra $f_k(\omega)$. If each $f_k(\omega)$ can be modeled as $f_k(\omega) \sim C_k\omega^{-\alpha_k}$ where $C = \mathbb{E}(C_k)$ and $\alpha = \mathbb{E}(\alpha_k)$, then $\log f_k(\omega) \sim \log C_k - \alpha_k \log \omega$ and an estimate of C and α can be obtained by finding the best linear approximation to all spectra in a neighborhood around $\omega = 0$. It has been shown that this estimate is influenced by the choice of this neighborhood (Andrews and Guggenberger, 2003; Hurvich and Deo, 1999). A simple extension to FBAM is shown here to automatically select this neighborhood as well as obtain estimates of C and α and therefore of $H = (1 - \alpha)/2$.

FBAM is extended to this task by constructing a piecewise *linear* approximation, as opposed to a piecewise *constant* approximation, to a collection of K power spectra on the log-log scale. That is, we seek a approximation to all K log spectra of the form

$$\log f_k(\omega) \approx \beta_0 + \beta_1 \log \omega + \sum_{\ell=1}^{L-1} b_\ell (\log \omega - \kappa_\ell)_+$$

where $(\log \omega - \kappa_\ell)_+ = \log \omega - \kappa_\ell$ if $\log \omega - \kappa_\ell > 0$ and $(\log \omega - \kappa_\ell)_+ = 0$ otherwise. This is a linear spline

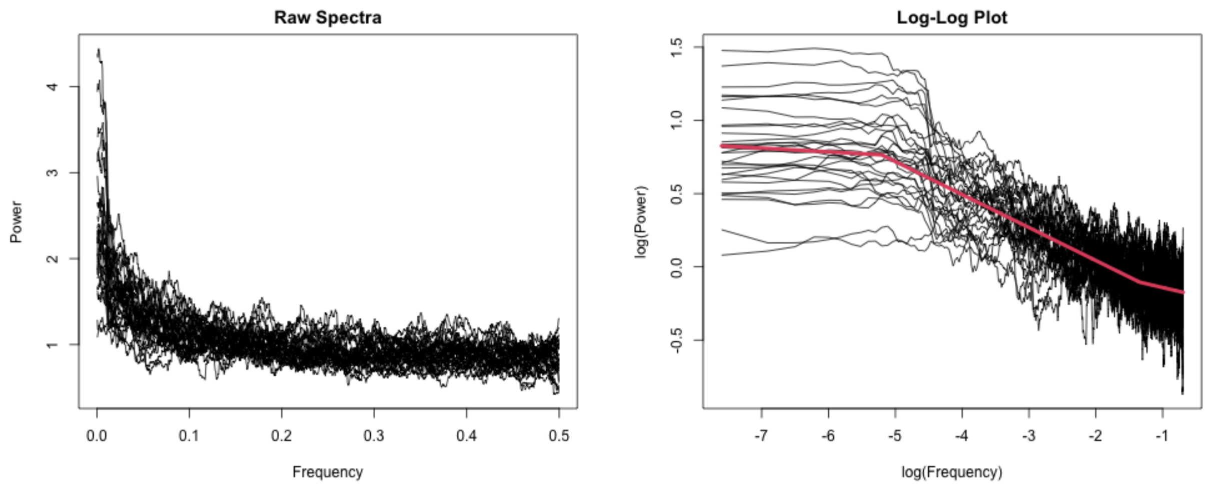

Figure 3: (Left) Raw multitaper estimated power spectra of a collection of $K = 30$ fractional ARIMA processes of length $T = 2000$. (Right) Spectra of the left panel on the log-log scale with the piecewise linear approximation estimated by our modified GA in red. Notice that the first frequency band corresponds to frequencies where the log spectra is mostly flat.

constructed from a linear spline basis with $L - 1$ knots located at $\kappa_1, \ldots, \kappa_{L-1}$. Choosing $L - 1$ knots is equivalent to specifying L frequency bands. Our extension automatically selects the locations of the knots for a given L using the genetic algorithm described in the manuscript with minor changes to simultaneously estimate the κ_ℓ, β_0, β_1, and $b_1, \ldots, b_L$. An objective of future research would be to develop a data-driven approach for choosing the number of knots L in this context.

We consider the following simulated example as a proof of concept of this extension. For $K \in \{30, 50, 100\}$ and $T \in \{500, 1000, 2000\}$, repeat the following 100 times. Simulate K fractional ARIMA processes of length T with Hurst exponents $H_k \sim U(0.55, 0.65)$. Note that $H = \mathbb{E}(H_k) = 0.6$ Our extended GA is then used to obtain estimates $\hat{\kappa}_1, \ldots, \hat{\kappa}_{L-1}$, $\hat{\beta}_0$, $\hat{\beta}_1$, and $\hat{b}_1, \ldots, \hat{b}_{L-1}$ with $L = 3$. Since log-power in ultra-low frequencies can bias estimates of the slope and consequently those of the Hurst parameter, we extract the estimated slope across frequencies from the second frequency band, $\hat{\alpha} = \hat{\beta}_1 + \hat{b}_1$. The estimated population-level Hurst parameter is then $\hat{H} = (1 - \hat{\alpha})/2$. Figure 3 displays the raw and log-log plots of multitaper estimated power spectra of a sample of $K = 30$ fractional ARIMA processes simulated as described above. The right panel of Figure 3 displays the piecewise linear approximation estimated by our modified GA. Table 3 displays the mean and SD of $\hat{H}$ across the 100 independent repetitions of this simulated example for each combination of K and T. We see that as T increases for any fixed K_j, we achieve a better estimate of the marginal Hurst parameter, $H = 0.6$.

K	T	$\hat{H}$
30	500	0.6261 (0.1783)
	1000	0.6246 (0.1087)
	2000	0.6225 (0.0377)
50	500	0.6227 (0.0562)
	1000	0.6249 (0.0558)
	2000	0.6136 (0.0164)
100	500	0.6265 (0.024)
	1000	0.6189 (0.0166)
	2000	0.6123 (0.0157)

Table 3: Mean (SD) of estimated marginal Hurst parameter for 100 independent replications of a collection of fractional ARIMA processes with replicate-specific Hurst parameters distributed uniformly over $(0.55, 0.65)$.

References

A. T. Walden. A unified view of multitaper multivariate spectral estimation. *Biometrika*, 87(4):767–788, December 2000. ISSN 0006-3444, 1464-3510. doi: 10.1093/biomet/87.4.767. URL `https://academic.oup.com/biomet/article-lookup/doi/10.1093/biomet/87.4.767`.

D.J. Thomson. Spectrum estimation and harmonic analysis. *Proceedings of the IEEE*, 70(9):1055–1096, 1982. ISSN 0018-9219. doi: 10.1109/PROC.1982.12433. URL `http://ieeexplore.ieee.org/document/1456701/`.

Donald B. Percival and Andrew T. Walden. *Spectral analysis for univariate time series.* Number 51 in Cambridge series on statistical and probabilistic mathematics. Cambridge University Press, Cambridge, 2020. ISBN 978-1-107-02814-2 978-1-139-23572-3. doi: 10.1017/9781139235723.

Christopher T. H. Baker. On the nature of certain quadrature formulas and their errors. *SIAM Journal on Numerical Analysis*, 5(4):783–804, December 1968. ISSN 0036-1429, 1095-7170. doi: 10.1137/0705059. URL `http://epubs.siam.org/doi/10.1137/0705059`.

Sanghamitra Bandyopadhyay. Genetic algorithms for clustering and fuzzy clustering. *WIREs Data Mining and Knowledge Discovery*, 1(6):524–531, November 2011. ISSN 1942-4787, 1942-4795. doi: 10.1002/widm.47. URL `https://wires.onlinelibrary.wiley.com/doi/10.1002/widm.47`.

A.E. Eiben and J.E. Smith. *Introduction to Evolutionary Computing.* Natural Computing Series. Springer Berlin Heidelberg, Berlin, Heidelberg, 2015. ISBN 978-3-662-44873-1 978-3-662-44874-8. doi: 10.1007/978-3-662-44874-8. URL `https://link.springer.com/10.1007/978-3-662-44874-8`.

Mimi Zhang and Andrew Parnell. Review of clustering methods for functional data. *ACM Transactions on Knowledge Discovery from Data*, 17(7):1–34, August 2023. ISSN 1556-4681, 1556-472X. doi: 10.1145/3581789. URL https://dl.acm.org/doi/10.1145/3581789.

Guilllermo Granados-Garcia, Mark Fiecas, Shahbaba Babak, Norbert J. Fortin, and Hernando Ombao. Brain waves analysis via a non-parametric Bayesian mixture of autoregressive kernels. *Computational Statistics & Data Analysis*, 174:107409, October 2022. ISSN 01679473. doi: 10.1016/j.csda.2021.107409. URL https://linkinghub.elsevier.com/retrieve/pii/S0167947321002437.

Donald W. K. Andrews and Patrik Guggenberger. A Bias-Reduced Log-Periodogram Regression Estimator for the Long-Memory Parameter. *Econometrica*, 71(2):675–712, March 2003. ISSN 0012-9682, 1468-0262. doi: 10.1111/1468-0262.00420. URL http://doi.wiley.com/10.1111/1468-0262.00420.

Clifford M. Hurvich and Rohit S. Deo. Plug-in Selection of the Number of Frequencies in Regression Estimates of the Memory Parameter of a Long-memory Time Series. *Journal of Time Series Analysis*, 20(3):331–341, May 1999. ISSN 0143-9782, 1467-9892. doi: 10.1111/1467-9892.00140. URL https://onlinelibrary.wiley.com/doi/10.1111/1467-9892.00140.
